# Mental health profiles of Finnish adolescents before and after the peak of the COVID-19 pandemic

**DOI:** 10.1186/s13034-023-00591-1

**Published:** 2023-04-29

**Authors:** Jasmine Gustafsson, Nelli Lyyra, Inga Jasinskaja-Lahti, Nina Simonsen, Henri Lahti, Markus Kulmala, Kristiina Ojala, Leena Paakkari

**Affiliations:** 1 Faculty of Sport and Health Sciences, University of Jyvaskyla, Jyvaskyla, Finland; 2 Faculty of Social Sciences, University of Helsinki, Helsinki, Finland; 3 Public Health Research Program, Folkhalsan Research Center, Helsinki, Finland; 4 Department of Public Health, University of Helsinki, Helsinki, Finland

**Keywords:** Mental health, Social relationships, Adolescence, COVID-19 pandemic, Cluster analysis

## Abstract

**Background:**

The COVID-19 pandemic has had implications for adolescents’ interpersonal relationships, communication patterns, education, recreational activities and well-being. An understanding of the impact of the pandemic on their mental health is crucial in measures to promote the post-pandemic recovery. Using a person-centered approach, the current study aimed to identify mental health profiles in two cross-sectional samples of Finnish adolescents before and after the peak of the pandemic, and to examine how socio-demographic and psychosocial factors, academic expectations, health literacy, and self-rated health are associated with the emerging profiles.

**Methods and findings:**

Survey data from the Health Behaviour in School-aged Children (HBSC) study conducted in Finland in 2018 (N = 3498, age *M* = 13.44) and 2022 (N = 3838, age *M* = 13.21) were analyzed. A four-profile model using cluster analysis was selected for both samples. In Sample 1, the identified profiles were (1) “Good mental health”, (2) “Mixed psychosocial health”, (3) “Somatically challenged”, and (4) “Poor mental health”. In Sample 2, the identified profiles were (1) “Good mental health”, (2) “Mixed psychosomatic health”, (3) “Poor mental health and low loneliness”, and (4) “Poor mental health and high loneliness”. The results of the mixed effect multinomial logistic regression analysis showed that in both samples, being a girl and reporting lower maternal monitoring; lower family, peer, and teacher support; higher intensity of online communication; a less positive home atmosphere and school climate; and poor self-rated health were most strongly linked to belonging to a poorer mental health profile. In addition, in Sample 2, low subjective health literacy was a key factor associated with poorer mental health profiles, and teacher support was more important than before COVID.

**Conclusions:**

The current study stresses the importance of identifying those vulnerable to developing poor mental health. To maximize post-pandemic recovery, the role of schools, especially teacher support and health literacy, along with the factors that remained important over time should be taken into account in public health and health promotion interventions.

**Supplementary Information:**

The online version contains supplementary material available at 10.1186/s13034-023-00591-1.

## Introduction

Research during the COVID-19 pandemic has raised concerns over the poor mental health of children and adolescents. According to several reviews and meta-analyses, the mental health of youths has deteriorated during the pandemic [1–4], particularly in terms of increased anxiety and depression symptoms. For example, the meta-analysis by Panda et al. [2] found that during the 1 year of the pandemic, between 31 and 42% of children and adolescents experienced mental health problems such as anxiety, depression, and irritability. In addition, a population-based longitudinal study in Germany [5] found that adolescents’ psychosomatic complaints were more prevalent during the pandemic compared to the pre-pandemic period: 23% of adolescents reported feeling low weekly before the pandemic, as compared with 34–43% in three waves during the first and second year of the pandemic. As the pandemic has impacted different population groups unequally [6], including adolescents [7, 8], it is important to identify the characteristics of the groups at particular risk of mental health problems and those who are more resilient to the adverse impact of the pandemic.

The deterioration of adolescents’ mental health could be partly explained by the different measures implemented to reduce the spread of the COVID-19 virus, such as social distancing, home quarantines, and remote schooling. Many of these measures restricted contact with other people, both peers and adults outside the home, as well as social support (e.g., perceptions of having someone who listens and encourages when needed) [9], resulting in detrimental effects on adolescents’ ability to fulfill their social needs and developmental tasks [10]. Given that social support may serve as a buffering mechanism between stressful events like the COVID-19 pandemic and poor mental health [11], adolescents with limited social support may have been especially vulnerable to the negative impacts of the pandemic. It has also been suggested that the effects of social distancing might extend beyond the pandemic [12], and that the dynamic of supportive relationships might have changed during this time period [13].

The effects of the pandemic during adolescence are not limited to peer relationships, they may also have an impact on the quality of relationships between parents and their children [14], which in turn can contribute to the overall health of adolescents [15]. During the pandemic, adolescents have reported lower levels of parental support than 6 months before the lockdown [16]. Furthermore, Magson et al. [17] observed that about a quarter of adolescents reported more frequent conflicts with their parents during the pandemic, which in turn was associated with lower life satisfaction. Families have also faced financial hardships during the pandemic, with parents from low-income and lower-middle class families being at greater risk of reduced income and job loss [18]. This could have affected the health of youths, as associations between low socioeconomic status and child mental health problems have received much support (for review, see Reiss [19]. Simultaneously, many adolescents have had less opportunities to interact with classmates and teachers. This is concerning, as teachers and the classroom environment have shown to play a vital role in supporting the well-being of young people [20, 21]. Since the COVID-19 pandemic began, adolescents have reported a decrease in communication with teachers and less emotional support from teachers (e.g., teachers listening to worries and concerns with less care), as measured during the fall of 2020 [22]. However, one study [23] has found that connectedness with school peers did not predict mental health among children and early adolescents during the pandemic in the spring and summer of 2020. This could possibly be due to the fact that relationships with other sources (e.g., family) may have played a more important role when access to classmates and teachers was limited.

New opportunities to interact and obtain social support have also arisen during the pandemic, with many adolescents spending more time with their friends online in spaces such as social media to compensate for the loss of face-to-face social interactions [24]. Turning to social media to talk with others can be one way of coping with the crisis [25]. However, systematic reviews, along with cross-national and single country studies, have indicated problematic social media use, including addiction-like symptoms (e.g., conflict with family and displacement of other activities due to social media use [26]), to associate with a multitude of psychological problems such as depressive symptomatology [27–30]. Moreover, it has been suggested that digitally mediated social interactions are not the same as face-to-face experiences, as spending more virtual time with friends during the pandemic has been associated with higher levels of depression among adolescents [31].

At the beginning of the pandemic, education practices changed, and online education increased. This may have affected adolescents’ school engagement and motivation, with evidence showing lower learning concentration, engagement, and ability to learn during online classes than in classroom learning [32]. These experiences, in turn, might have an impact on adolescents’ plans for future education, which have also been linked to health. For example, lower educational expectations (e.g., expecting an education lower than university) have been associated with poorer mental health in terms of higher levels of externalizing problems (e.g., hyperactivity) among youths [33].

In addition to different psychosocial assets, other individual assets, such as health literacy, could also serve as a buffering mechanism against the negative effects of the pandemic on adolescents’ mental health. During the pandemic, the role of health literacy as a set of competencies (e.g., knowledge on health issues and an ability to seek and assess information) necessary for promoting and sustaining one’s health and that of others [34] has grown in importance. It has been important for following safety regulations, for seeking timely help and for finding valid health information from among the massive flow of information of different quality provided on the internet in particular. Low health literacy has been linked with not only difficulties in understanding COVID-19 information and infection prevention behaviors, but also with poorer mental health [35]. Already before the pandemic, low health literacy was recognized as an independent explanatory factor in mental health variance (e.g., feeling low) [36].

The pandemic has also had adverse effects on adolescents’ self-rated health, that is, a person’s overall health status [37], especially among those with limited social support [38]. This is disturbing, given that poorer self-rated health during adolescence has been linked to health problems in adulthood [39] and has shown to be a robust predictor of mortality [40]. According to pre-pandemic research, associations between higher stress caused by uncertainty and poorer self-rated health have also been observed [41], whereas better self-rated health has been linked to better mental health in terms of lower anxiety [42] and higher resilience [43], that is, the ability to maintain one’s mental health when facing adversity [44]. For these reasons, self-rated health could be an important factor in how adolescents react or adapt to the stressors caused by the pandemic.

The different effects of the pandemic on different individuals may also be due to their socio-demographic characteristics. A vast body of research has shown that girls already reported poorer mental health in terms of depression than boys prior to the pandemic (for a meta-analysis, see Salk et al. [45]). Pre-pandemic research [46, 47] has also linked other characteristics of young people, such as older age and immigrant status, to poorer mental health (e.g., depression, anxiety). Studies conducted in different parts of the world have shown that youths living in urban areas tend to report slightly poorer well-being than those living in rural areas [48, 49], although country-specific differences exist. These individuals may be particularly vulnerable to the adverse effects of the pandemic.

### The pandemic in the Finnish context

Beginning in mid-March 2020, Finnish schools were closed nationwide for about two months [50]. During fall 2020 and spring 2021, education was temporarily conducted in the form of distance learning in some regions, especially upper education [51]. From fall 2021 until spring 2022, Finnish comprehensive schools remained fully open. Since the onset of the COVID-19 pandemic, leisure centers and sports facilities have closed several times, with closures lasting a few weeks to several months [52, 53]. Most sports facilities have remained open since February 2022 [54]. As in many countries, mental health problems have increased among Finnish adolescents during the pandemic. In spring 2021, satisfaction with life had decreased, while anxiety, depression and feelings of loneliness had increased from 2019 [55, 56]. However, it should be noted that already during the last two decades prior to the pandemic, Finnish adolescents’ psychological and somatic health complaints (e.g., depression and headaches) had increased [57].

In sum, the pandemic has affected the lives of youths and their families in many ways. Understanding the implications of the COVID-19 pandemic for the mental health of youths and the potential risk and protective factors is crucial for measures to promote post-pandemic recovery. To date, most studies examining the impact of the pandemic on the mental health of adolescents have focused on the prevalence of symptoms of depression or anxiety and used variable-centered approaches aimed at predicting their different mental health outcomes, with findings largely relying on data collected during the 1 year of the pandemic. The mental health outcomes of survivors of the pandemic may be highly individual and linked to different psychosocial and health assets or resources, including primary and institutionalized support systems such as families and school. To show this diversity of mental health reactions and to identify vulnerabilities to the pandemic, in this study, we adopted a person-centered approach, that is, statistical techniques that identify groups of individuals who share particular characteristics that are similar within groups but different between groups [58]. Drawing on two large data sets of repeated cross-sectional design—the first at 2 years prior to COVID-19 and the second at 2 years after the beginning of the pandemic—the current study aimed to identify mental health profiles among Finnish adolescents before (2018) and after the peak of the pandemic (2022), and then to analyze which socio-demographic, psychosocial, and other health-related factors characterize adolescents who are at risk and those who are more resilient to the detrimental impact of the pandemic on mental health.

The specific aims of this study were:

1. To identify mental health profiles (psychological complaints, somatic complaints, life satisfaction, perceived loneliness, and problematic social media use) among Finnish adolescents before and after the peak of the COVID-19 pandemic
2. To examine how socio-demographic characteristics (gender, age, language of instruction, immigrant background, family affluence, family structure, urban/rural residence), psychosocial factors (perceived social support, perceived home atmosphere, parental monitoring, perceptions of school climate, intensity of online communication), educational expectations, health literacy, and self-rated health are associated with the health profiles before and after the peak of the pandemic.

## Methods

### Participants and procedure

Data were collected from two cross‐sectional samples of Finnish adolescents in the 5th, 7th, and 9th grades in 2018 (N = 3498) and 2022 (N = 3838), as part of the Health Behaviour in School-aged Children (HBSC) study. The HBSC study is carried out in collaboration with the World Health Organization (WHO) Regional Office for Europe. Samples were drawn using a cluster sampling method, with schools as the primary sampling unit. The sampling was adjusted for province, municipality, and school size. The collection of data followed the protocol of the international HBSC study, ensuring responsible conduct of research [59]. The respondents answered the online surveys during the school day in the spring semester. Participation was voluntary and no personally identifiable information was collected. The surveys were approved by the Ethical Committee of the University of Jyvaskyla.

### Measures

Table 1 presents the study variables. Mental health indicators were psychological complaints, somatic complaints, life satisfaction, perceived loneliness, and problematic social media use. Information on socio-demographic characteristics (immigrant background, family affluence, family structure, urban/rural residence), psychosocial factors (perceived social support, perceived home atmosphere, parental monitoring, perceptions of school climate, intensity of online communication), educational expectations, health literacy, and self-rated health was also collected. The adolescents also reported their gender (1 = Boy, 2 = Girl). Grade level consisted of the following categories: 1 = 5th grade (age *M* = 11.39 in Sample 1/11.38 in Sample 2), 2 = 7th grade (age *M* = 13.37 in Sample 1/13.52 in Sample 2), and 3 = 9th grade (age *M* = 15.33 in Sample 1/15.37 in Sample 2). The language of instruction, which partly served as an indicator of group status, was based on the teaching language of the schools (1 = Finnish, 2 = Swedish), as Finland is a bilingual country with two official languages, Finnish being the mother tongue of the majority and Swedish that of the linguistic minority. A detailed description of the instruments of the HBSC survey can be found in the HBSC Study Protocol by Inchley et al. [59].

**Table 1:** Measures used in the study

Variable and items	Response options	Coding	Scale used/adapted from	Cronbach's alpha	
				Sample 1	Sample 2
Mental health indicators					
Psychological complaints					
How often have you experienced these symptoms over the last six months?	About every day More than once a week About every week About every month Rarely or never	Number of complaints (1 = 0, 2 = 1–2, 3 = 3–4) experienced twice a week or more often	HBSC symptoms checklist [60]. The measure has proven validity [60]	0.83	0.82
Somatic complaints					
How often have you experienced these symptoms over the last six months?	About every day More than once a week About every week About every month Rarely or never	Number of complaints (1 = 0, 2 = 1–2, 3 = 3–4) experienced twice a week or more often	HBSC symptoms checklist [60]. The measure has proven validity [60]	0.75	0.76
Life satisfaction					
Adolescents were asked to rate how satisfied they were with their lives on a visual scale	10 The best possible life—0 The worst possible life	The scale was reversed and used as a continuous scale (0–10)	Cantril ladder [61]. The measure has shown good test–retest reliability and convergent validity [62]		
Perceived loneliness					
Do you ever feel lonely? (Sample 1)	Yes, very often Yes, quite often Sometimes No	1 = Low loneliness (No / Sometimes), 2 = High loneliness (Yes, quite often / Yes, very often)			
How often have you felt lonely during the last 12 months? (Sample 2)	Never Rarely Sometimes Most of the time Always	1 = Low loneliness (Never / Rarely / Sometimes), 2 = High loneliness (Most of the time /Always)			
Problematic social media use					
During the past year, have you	No Yes	All yes-responses were summed (range 0–9) and recoded into three groups: normative users (0 to 1 yes-responses), risky users (2 to 5 yes-responses), and problematic users (6 to 9 yes-responses) [30, 63]	Social Media Disorder Scale [26]. The measure has proven validity [63]	0.82	0.82
Regularly noticed that you can only think about the moment that you will be able to use social media again?					
Regularly felt dissatisfied because you wanted to spend more time on social media?					
Often been in a bad mood because you couldn’t use social media?					
Tried to spend less time on social media but failed?					
Regularly neglected doing other things (e.g., hobbies, sports) because you wanted to use social media?					
Regularly argued with others about your own social media usage?					
Regularly lied to your parents or friends about how much time you spend on social media?					
Often used social media to escape from unpleasant feelings?					
Actually fought with your parents or siblings because of your social media usage?					
Psychosocial factors					
Perceived home atmosphere					
How would you rate the atmosphere in your home?	Very good Quite good Neither good nor bad Quite bad Very bad	The scale was reversed and used as a continuous variable	Simonsen et al. [64]; Suominen et al. [65]		
Parental monitoring					
Maternal monitoring (6 items)^a^			Brown [66]	0.86	0.86
Paternal monitoring (6 items)^a^			Brown [66]	0.89	0.90
How much does your mother/father really know about	She/he knows a lot She/he knows something She/he knows nothing I have no mother, or I do not see her I have no father, or I do not see him	The last two response options were omitted. Mean scores were calculated for both subscales			
Who your friends are					
How you spend your money					
Where you spend your time after school					
Where you are in the evenings					
What you do in your free time					
What you do on the internet					
Family support					
My family really tries to help me	Very strongly disagree (1)—Very strongly agree (7)	Items were computed into a mean score	Multi-dimensional Scale of Perceived Social Support [67]. The measure has proven validity [68]	0.97	0.96
I get the help and emotional support I need from my family					
I can talk about my problems with my family					
My family is willing to help me in decision-making					
Peer support					
My friends really try to help me	Very strongly disagree (1)— Very strongly agree (7)	Items were computed into a mean score	Multi-dimensional Scale of Perceived Social Support [67]. The measure has proven validity [68]	0.96	0.96
I can count on my friends when something goes wrong					
I have friends with whom I can share my joys and sorrows					
I can talk about my problems with my friends					
Teacher support					
I feel that my teachers accept me as I am	Completely agree Agree Neither agree nor disagree Disagree Completely disagree	Items were reversed and computed into a mean score	Teacher and Classmate Support Scale [69]. The measure has proven validity [70]	0.87	0.88
I feel that my teachers care about me as a person					
I trust my teachers a lot					
Classmate support					
The pupils in my class get on well with each other	Completely agree Agree Neither agree nor disagree Disagree Completely disagree	Items were reversed and computed into a mean score	Teacher and Classmate Support Scale [69]. The measure has proven validity [70]	0.81	0.84
Most pupils in my class are kind and helpful Other pupils accept me the way I am					
School climate					
I feel safe in this school	Completely agree Agree Neither agree nor disagree Disagree Completely disagree	Items were reversed and computed into a mean score	The School Climate Index [71]	0.81	0.86
I feel like I belong in this school					
Intensity of online communication					
How often do you interact with the following people via the internet?	I don’t know/Doesn’t concern me Never or hardly ever At least every week Daily or almost daily Several times a day Almost all the time	The first response option was omitted. Following previous research [72, 73], all items were calculated into a mean score	EU Kids Online Survey [74]	0.71	0.74
Close friend(s)					
Friends from a larger friend group					
People that you have met on the internet					
Other people (e.g., parents, siblings, classmates, teachers)					
Other health-related factors					
Educational expectations^b^					
What will you do when you finish comprehensive school?	Sample 1: Apply for general upper secondary education Apply to a vocational school or for other vocational training Apply for an apprenticeship Double qualification *(e.g., general upper secondary education and vocational upper secondary education simultaneously)* Get a job Remain unemployed I don’t know Sample 2: Apply for general upper secondary education Apply to a vocational school or for other vocational training Double qualification *(e.g., general upper secondary education and vocational upper secondary education simultaneously)* Apply for voluntary additional education (e.g., 10^th^ grade) I don’t know	Responses were categorized into 1 = academic educational expectations (upper secondary school) and 2 = vocational educational expectations (vocational school or other vocational training). Other response options were omitted due to their low frequency (< 5%)			
Health literacy^a^					
I am confident that …	Not at all true Not completely true Somewhat true Absolutely true	A sum score was calculated (range 0–40), and further recoded into three groups: low (score 10–25), moderate (26–35) and high health literacy (36–40) [75]	Health Literacy for School-Aged Children (HLSAC) instrument [76]. The measure has proven validity [76]	0.96	0.92
I have good information about health					
When necessary, I am able to give ideas on how to improve health in my immediate surroundings (e.g., a nearby place or area, family, friends)					
I can compare health-related information from different sources					
I can follow the instructions given to me by healthcare personnel (e.g., nurse, doctor)					
I can easily give examples of things that promote health					
I can judge how my own actions affect the surrounding natural environment					
When necessary, I can find health-related information that is easy for me to understand					
I can judge how my behavior affects my health					
-I can usually figure out if some health-related information is right or wrong					
I can give reasons for the choices I make regarding my health					
Self-rated health					
Would you say your health is…?	Excellent Good Fair Poor	The scale was reversed and used as a continuous variable			
Socio-demographic characteristics					
Family affluence					
Does your family own a car (a passenger car, a van, or a lorry)?	No One Two or more	A sum score (range 0–10) was calculated from the items, which was further categorized into three groups of relative family affluence: low (lowest 20th percentile), medium (between 20 and 80th percentile) and high (highest 20th percentile), in line with international guidelines [77]. Due to the distribution of the sum score, the following cut-offs were used: 0–6 (low, lowest 18th percentile), 7–8 (medium, between 19th and 73rd percentile) and 9–10 (high, highest 27th percentile)	HBSC Family Affluence Scale (FAS III) [78]. The measure has been shown to correlate moderately with parental earned income [79]		
Do you have your own bedroom?	No Yes				
How many computers does your family have (including laptops and tablets, but not game consoles and smartphones)?	None One Two More than two				
How many bathrooms are in your home?	None One Two More than two				
Do you have a dishwasher in your home?	No Yes				
		Family structure			
Answer this question by thinking about the home in which you live all the time or most of the time, and mark the people who live there	Mother Father Mother’s partner Father’s partner I live in a foster home or children’s home Someone or somewhere else (e.g., grandparents)	Responses were categorized into three family structures (1 = lives in a nuclear family, 2 = lives in a single-parent family, 3 = lives in a step-family). Cases in which the adolescents either left the question blank (*n* = 33 in sample 1, *n* = 41 in sample 2) or reported living without a parent (*n* = 22 in sample 1, *n* = 27 in sample 2) were set to missing due to the low frequency of those responses			
Urban/rural residence					
What kind of place do you live in?	City, in the center City, outside the center Countryside, in the village center Countryside, outside the village center	Responses were dichotomized into 1 = urban residence (City, in the center / City, outside the center) and 2 = rural residence (Countryside, in the village center / Countryside, outside the village center)			
Immigrant background					
This variable was constructed from three items asking in which country the adolescents and each of their parents were born	Lists of countries	Responses were categorized into 1 = first-generation immigrant (being born abroad), 2 = second-generation immigrant (born in Finland and one or both parents born abroad), and 3 = native/non-immigrant background (the respondent and parent(s) were born in Finland)			

^a^answered only by 7th and 9th grade adolescents

^b^answered only by 9th grade adolescents

### Statistical analysis

Differences between the key variables in Samples 1 and 2 were analyzed using Chi-square tests and independent *t*-tests, with Bonferroni-corrected *p*-values for multiple testing. Correlations were calculated using Spearman's rank correlation, and differences between the correlations of samples were compared using Fisher r-to-z transformations. Mental health profiles, based on five indicators (i.e., psychological complaints, somatic complaints, life satisfaction, perceived loneliness, and problematic social media use), were identified separately for Samples 1 and 2 using the SPSS TwoStep Clustering algorithm. This exploratory method identifies subgroups of adolescents based on similarities in their characteristics. The number of clusters were allowed to be automatically estimated by the analysis method on the basis of the Bayesian information criterion (BIC), and various fixed numbers of clusters were also tested. Missing data were handled using listwise deletion, and differences in socio-demographic characteristics between included and excluded cases were examined further using Chi-square test, analysis of variance (ANOVA), post-hoc ANOVA analysis with Bonferroni correction, and Generalized linear mixed models with multinomial logistic regression. The final cluster solution was determined on the basis of cluster quality (silhouette coefficient), size, and interpretability. The Chi-square test, ANOVA, and post-hoc ANOVA analysis with Bonferroni correction were used to compare the clusters. The clusters were named on the basis of the interpretation of the most notable characteristics that made up the profiles.

Generalized linear mixed models with multinomial logistic regression were performed separately for the two samples, to assess the associations between independent variables and mental health profiles, using the “Good mental health” profile as the reference category. A multilevel analysis with “school” included as a random effect was chosen on the basis of the structure of the data, as the adolescents were nested within schools. No multicollinearity was detected among the independent variables (Variance Inflation Factor (VIF) < 2 in both samples). Crude odds ratios were calculated for all the independent variables. Two adjusted models were performed: the first included all the socio-demographic characteristics, and the second included all the independent variables. A separate analysis of only 7th and 9th grade adolescents was performed for parental monitoring and health literacy, as these variables were only measured in these grades. In addition, a separate analysis of the educational expectations of 9th grade adolescents only was conducted, as these were only measured in this grade. Missing data were handled using listwise deletion. Data were analyzed using IBM SPSS Statistics 28.0.

## Results

### Sample characteristics

Table 2 presents the frequencies, means and standard deviations of the socio-demographic characteristics and key variables of the two samples separately. In both samples, about half were girls (50/51%) and lived in an urban area (55/57%), and the majority had Finnish as their language of instruction (80/63%), lived in a nuclear family (75/69%), and had a native (non-immigrant) background (88/89%). Compared to Sample 1, the adolescents in Sample 2 had a lower mean age (*M* = 13.44/13.21 years).

**Table 2:** Comparison of study variables in Samples 1 and 2

	Sample 1 (2018), *N* = 3498	Sample 2 (2022), *N* = 3838	Significance
	% (*n*)/*M* (*SD*)	% (*n*)/*M* (*SD*)	
Socio-demographic characteristics			
Gender, female (vs. male)	49.9 (1726)	50.6 (1915)	χ^2^ = 0.39, *p* = 0.531
Mean age	**13.44 (1.69)**	**13.21 (1.74)**	**t(7276) = 5.75, *p* < 0.001**
Grade			χ^2^**= 68.12, *p* < 0.001**
5th	**29.8 (1041)**	**37.3 (1432)**	***p* < 0.001**^d^
7th	36.6 (1281)	36.8 (1413)	*p* > 0.05^d^
9th	**33.6 (1176)**	**25.9 (993)**	***p* < 0.001**^d^
Language of instruction, Swedish (vs. Finnish)	**19.8 (691)**	**37.1 (1424)**	χ^2^** = 268.44, *p* < 0.001**
Relative family affluence			χ^2^= 0.42, *p* = 0.812
Low	17.8 (610)	17.9 (680)	*p* > 0.05^d^
Medium	56.0 (1919)	55.4 (2108)	*p* > 0.05^d^
High	26.1 (895)	26.8 (1019)	*p* > 0.05^d^
Family structure			χ^2^**= 65.34, *p* < 0.001**
Nuclear family	**74.7 (2508)**	**69.3 (2313)**	***p* < 0.001**^d^
Single-parent family	**13.6 (457)**	**21.0 (700)**	***p* < 0.001**^d^
Step-family	**11.7 (391)**	**9.7 (323)**	***p* = 0.009**^**d**^
Urban residence (vs. rural)	55.2 (1911)	57.1 (2152)	χ^2^= 2.77, *p* = 0.096
Immigrant background			χ^2^= 0.79, *p* = 0.675
First-generation immigrant	4.5 (156)	4.2 (154)	*p* > 0.05^d^
Second-generation immigrant	7.2 (247)	6.9 (253)	*p* > 0.05^d^
Native (non-immigrant)	88.2 (3026)	88.9 (3256)	*p* > 0.05^d^
Mental health indicators			
Psychological complaints^a^			χ^2^**= 32.60, *p* < 0.001**
0	**57.5 (1986)**	**51.2 (1927)**	***p* < 0.001**^d^
1–2	**27.3 (942)**	**29.6 (1115)**	***p* = 0.026**^d^
3–4	**15.2 (525)**	**19.1% (719)**	***p* < 0.001**^d^
Somatic complaints^a^			χ^2^**= 28.74, *p* < 0.001**
0	**69.2 (2391)**	**64.4 (2421)**	***p* < 0.001**^d^
1–2	**26.2 (903)**	**28.5 (1073)**	***p* = 0.023**^d^
3–4	**4.6 (159)**	**7.1 (266)**	***p* < 0.001**^d^
Life satisfaction	**7.72 (1.81)**	**7.42 (1.67)**	**t(6847) = 7.28, *p* < 0.001**
High loneliness (vs. low)	**14.8 (503)**	**11.2 (420)**	χ^2^** = 20.48, *p* < 0.001**
Problematic social media use			χ^2^**= 13.08, *p* < 0.001**
Normative user	**56.2 (1806)**	**52.0 (1737)**	***p* = 0.001**^d^
Risky user	**34.3 (1102)**	**38.5 (1284)**	***p* < 0.001**^d^
Problematic user	9.5 (307)	9.5 (318)	*p* > 0.05^d^
Psychosocial factors			
Perceived home atmosphere	4.31 (0.79)	4.33 (0.79)	t(6854) = − 0.94, *p* > 0.05
Parental monitoring^b^			
Maternal monitoring	**2.43 (0.45)**	**2.51 (0.44)**	**t(5773) = **− **5.99, *p* < 0.001**
Paternal monitoring	**2.24 (0.54)**	**2.33 (0.55)**	**t(5548) = **− **5.99, *p* < 0.001**
Family support	5.67 (1.67)	5.60 (1.66)	t(6859) = 1.80, *p* > 0.05
Peer support	5.42 (1.68)	5.45 (1.65)	t(6838) = − 0.79, *p* > 0.05
Teacher support	**3.84 (0.95)**	**3.96 (0.94)**	**t(6898) = **− **5.27, *p* < 0.001**
Classmate support	**3.90 (0.79)**	**3.79 (0.86)**	**t(6946) = 5.19, *p* < 0.001**
Perceived school climate	**4.14 (0.88)**	**3.97 (0.95)**	**t(6987) = 7.58, *p* < 0.001**
Intensity of online communication	3.02 (0.85)	3.05 (0.96)	t(6048) = − 0.92, *p* > 0.05
Other health-related factors			
Academic educational expectations^c^ (vs. vocational)	**64.71 (704)**	**58.47 (518)**	χ^2^**= 8.07,* p* = 0.005**
Health literacy^b^			χ^2^**= 15.00,* p* = 0.005**
Low	10.2 (238)	8.8 (208)	*p* > 0.05^d^
Moderate	**55.5 (1301)**	**61.1 (1446)**	***p* < 0.001**^d^
High	**34.3 (805)**	**30.2 (714)**	***p* = 0.002**^d^
Self-rated health			x**= 31.36, *p* < 0.001**
Poor	2.4 (84)	2.1 (79)	*p* > 0.05^d^
Fair	13.9 (479)	12.9 (487)	*p* > 0.05^d^
Good	**60.1 (2074)**	**55.7 (2106)**	***p* < 0.001**^d^
Excellent	**23.6 (813)**	**29.4 (1111)**	***p* < 0.001**^d^

Chi-square test for percentage comparison and independent t-test for mean comparison. Scores ranged from 10 to 19 for age, 0 to 10 for life satisfaction, 1 to 5 for home atmosphere, 1 to 3 for parental monitoring, 1 to 7 for family and peer support, 1 to 5 for teacher and classmate support, 1 to 5 for school climate, and 1 to 5 for intensity of online communication. Bold values denote statistical significance

^a^Number of complaints experienced more than once a week

^b^Only assessed among 7th and 9th grade adolescents (sample 1, *n* = 2457, sample 2, *n* = 2406)

^c^Only assessed among 9th grade adolescents (sample 1, *n* = 1176, sample 2, *n* = 993)

^d^Bonferroni-corrected *p*-values for multiple testing

Table 3 shows the correlations of mental health indicators in both samples. In both samples, all five mental health indicators correlated significantly (*p* < 0.001), with weak to moderate correlations ranging from 0.17 to 0.51. The Fisher r-to-z transformations showed that most of the correlations were significantly stronger in Sample 2 (*p*-values varied between < 0.001 and 0.035), with the exception of psychological complaints, which correlated more strongly with loneliness in Sample 1 (*p* = 0.004, see also Additional file 1: Table S1). The correlations between somatic complaints and loneliness, loneliness and life satisfaction, and loneliness and problematic social media use did not differ in the two samples (*p* > 0.05).

**Table 3:** Correlations of mental health indicators in both samples

	1	2	3	4
Sample 1 (2018)				
1 Psychological complaints^a^	–			
2 Somatic complaints^a^	0.45**	–		
3 Life satisfaction^b^	− 0.40**	− 0.26**	–	
4 Perceived loneliness^a^	0.37**	0.22**	− 0.33**	–
5 Problematic social media use^a^	0.26**	0.18**	− 0.22**	0.17**
Sample 2 (2022)				
1 Psychological complaints^a^	–			
2 Somatic complaints^a^	0.51**	–		
3 Life satisfaction^b^	− 0.46**	− 0.33**	–	
4 Perceived loneliness^a^	0.31**	0.25**	− 0.32**	–
5 Problematic social media use^a^	0.33**	0.23**	− 0.28**	0.19**

Spearman’s rank correlation

^**a**^Higher values indicate poorer mental health

^**b**^Higher values indicate higher life satisfaction

^**^p < 0.001

### Mental health profiles

#### Sample 1 (2018)

**Fig. 1:**
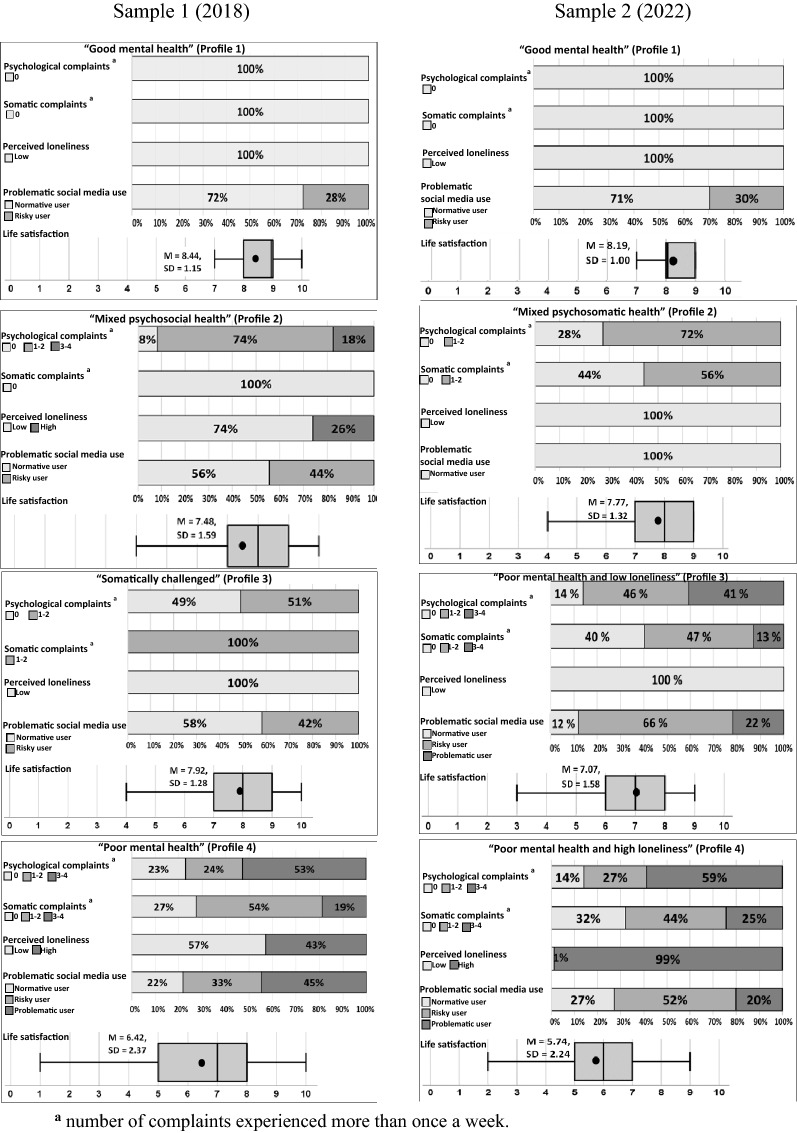
Stacked bar plots and box plots showing distribution of mental health indicators in each profile in both samples

Tables 4, 5 and Fig. 1 describe the results of the two-step cluster analysis in both samples. In Sample 1, 3149 responses (90%) of a potential 3498 responses were eligible for the cluster analysis, and there were small but significant variations in socio-demographic variables between those who were eligible for this analysis and those who were excluded (see Additional file 1: Tables S2, S3). As shown in mixed effect multinomial logistic regression analysis, those who were excluded were more likely to be boys (*p* < 0.001) and first-generation immigrants (*p* = 0.034), and to report lower family affluence (*p* < 0.001) compared to those who were included.

**Table 4:** Mental health profiles in sample 1 (2018)

	All	Profile 1	Profile2	Profile 3	Profile 4	Profile comparison		
	*n* = 3149	“Good mental health” (43.7%, *n* = 1375)	“Mixed psychosocial health” (19.9%, *n* = 628)	“Somatically challenged” (15.0%, *n* = 471)	“Poor mental health” (21.4%, *n* = 675)	Overall	*p*-value	Pairwise comparison
	%/*M* (*SD*)	%/*M* (*SD*)	%/*M* (*SD*)	%/*M* (*SD*)	%/*M* (*SD*)	χ^2^/*F*		
Sample 1 (2018)								
Mental health indicators								
Psychological complaints^a^						2747.51	< 0.001	
0	57.5	100.0	8.1	49.0	22.8			All profiles differ^b^
1–2	27.7		74.4	51.0	24.4			2, 3, 4 differ^b^
3–4	14.8		17.5		52.7			2, 4 differ^b^
Somatic complaints^a^						2919.90	< 0.001	
0	69.5	100.0	100.0		27.3			1 = 2, 4 differs^b^
1–2	26.5			100.0	53.8			3, 4 differ^b^
3–4	4.1				19.0			
Life satisfaction	7.74 (1.77)	8.44 (1.15)	7.48 (1.59)	7.92 (1.28)	6.42 (2.37)	251.64	< 0.001	All profiles differ^b^
High loneliness (vs. low)	14.5	0.0	26.4	0.0	43.0	827.20	< 0.001	1 = 3, 2^b^, 4 differ^b^
Problematic social media use						1363.10	< 0.001	
Normative user	56.1	72.4	55.6	58.2	21.8			2 = 3, others differ^b^
Risky user	34.3	27.6	44.4	41.8	33.3			2 = 3, others differ^c^
Problematic user	9.6				44.9			

Chi-square test for percentage comparison and Post-hoc ANOVA for mean comparison (two-tailed)

^a^Number of complaints experienced more than once a week

^b^Profiles differed significantly (*p* < 0.001) using Bonferroni-corrected pairwise comparisons

^c^Profiles differed significantly (*p* = 0.042) using Bonferroni-corrected pairwise comparisons

**Table 5:** Mental health profiles in Sample 2 (2022)

	All	Profile 1	Profile 2	Profile 3	Profile 4	Profile comparison		
	*n* = 2981	“Good mental health” (37.0%, *n* = 1103)	“Mixed psychosomatic health” (16.7%, *n* = 499)	“Poor mental health and low loneliness” (33.9%, *n* = 1011)	“Poor mental health and high loneliness” (12.3%, *n* = 368)	Overall	*p*-value	Pairwise comparison
	%/*M* (*SD*)	%/*M* (*SD*)	%/*M* (*SD*)	%/*M* (*SD*)	%/*M* (*SD*)	χ^2^/*F*		
Sample 2 (2022)								
Mental health indicators								
Psychological complaints^a^						2453.62	< 0.001	
0	48.1	100.0	27.7	13.9	14.1			3 = 4, others differ^b^
1–2	30.9		72.3	45.5	27.2			2, 3, 4 differ^b^
3–4	21.0			40.6	58.7			3, 4 differ^b^
Somatic complaints^a^						1264.00	< 0.001	
0	62.1	100.0	44.3	40.4	32.1			2 = 3, others differ^c^
1–2	30.6		55.7	46.9	43.5			3 = 4, 2 differs^d^
3–4	7.3			12.8	24.5			3, 4 differ^b^
Life satisfaction	7.44 (1.66)	8.19 (1.00)	7.77 (1.32)	7.07 (1.58)	5.74 (2.24)	293.31	< 0.001	All profiles differ^b^
High loneliness (vs. low)	12.2	0.0	0.0	0.0	98.9	2944.09	< 0.001	1 = 2 = 3, 4 differs^b^
Problematic social media use						1437.46	< 0.001	
Normative user	50.2	70.5	100.0	11.8	27.4			All profiles differ^b^
Risky user	39.9	29.5		66.4	52.4			All profiles differ^b^
Problematic user	9.9			21.9	20.1			3 = 4

Chi-square test for percentage comparison and Post-hoc ANOVA for mean comparison (two-tailed)

^a^Number of complaints experienced more than once a week

^b^Profiles differed significantly (*p* < 0.001) using Bonferroni-corrected pairwise comparisons

^c^Profiles differed significantly (*p* = 0.015) using Bonferroni-corrected pairwise comparisons

^d^Profiles differed significantly (*p* = 0.004) using Bonferroni-corrected pairwise comparisons

In Sample 1 (*n* = 3149), four profiles were identified and labeled as follows: 1) “Good mental health” (44%, *n* = 1375), 2) “Mixed psychosocial health” (20%, *n* = 628), 3) “Somatically challenged” (15%, *n* = 471), and 4) “Poor mental health” (21%, *n* = 675). The silhouette coefficient was 0.40, indicating fair cluster quality.

Adolescents in the “Good mental health” profile reported low prevalence (i.e., experienced no more often than once a week) of psychological and somatic complaints. They were highly satisfied with their lives (*M* = 8.44) and the majority (72%) were normative social media users. All adolescents in this profile reported low loneliness (100%).

The average life satisfaction of the adolescents in the “Mixed psychosocial health” profile was moderate (*M* = 7.48). This profile had the highest percentage of adolescents reporting one to two frequent (i.e., experienced more often than once a week) psychological complaints (74%), whereas the prevalence of somatic complaints was low. Moreover, roughly one fourth (26%) of the adolescents reported high loneliness and nearly half (44%) were risky social media users.

The “Somatically challenged” profile had the highest percentage (100%) of adolescents reporting one to two frequent somatic complaints. Around half (49%) reported low prevalence of psychological complaints and the rest reported one to two frequent psychological complaints. Their mean life satisfaction was 7.92, and all the adolescents reported low loneliness. The majority (58%) were normative social media users, and the rest (42%) were risky social media users.

The “Poor mental health” profile was the only one with adolescents who reported three to four frequent psychological (53%) or somatic complaints (19%) in Sample 1. This profile also had the highest percentage of adolescents reporting high loneliness (43%), the lowest mean value of life satisfaction (*M* = 6.42), and the highest percentage of problematic social media users (44%).

#### Sample 2 (2022)

In Sample 2, 2981 responses (78%) of a potential 3838 responses were acceptable for the cluster analysis, and there were small but significant differences in socio-demographic variables between those who were included in this analysis and those who were excluded (see Additional file 1: Tables S2, S3). As shown in mixed effect multinomial logistic regression analysis, those who were excluded were more likely to be boys (*p* < 0.001), and first- (*p* < 0.001) or second-generation immigrants (*p* = 0.002), and to have Swedish as opposed to Finnish as language of instruction (*p* = 0.010), and they were less likely to be in the 9th grade (*p* = 0.012) compared to those who were included.

In this sample (*n* = 2981), four profiles were observed: (1) “Good mental health” (37%, *n* = 1103), (2) “Mixed psychosomatic health” (17%, *n* = 499), 3) “Poor mental health and low loneliness” (34%, *n* = 1011), and 4) “Poor mental health and high loneliness” (12%, *n* = 368). The silhouette coefficient was 0.40, indicating fair cluster quality. Notably, the distribution of mental health indicators in the different profiles was quite similar for one profile in both samples (i.e., “Good mental health”). However, the proportion of adolescents belonging to this profile differed in the two samples, with fewer adolescents belonging to this profile in Sample 2 (2022) than in Sample 1 (2018) (*p* = *0.001*).

Adolescents in the “Good mental health” profile reported low prevalence of psychological and somatic complaints. The mean value of life satisfaction (*M* = 8.19) was the highest in this profile, and all adolescents reported low loneliness (100%). The majority were normative social media users (71%).

In the “Mixed psychosomatic health” profile, the majority experienced one to two frequent psychological (72%) or somatic complaints (56%). Their mean value of life satisfaction (*M* = 7.77) was moderate, and all the adolescents reported low loneliness (100%). This was the only profile in which all the adolescents were normative social media users (100%).

In the “Poor mental health and low loneliness” profile, most adolescents reported experiencing one to two (46%) or three to four (41%) frequent psychological complaints. Almost 60 percent experienced at least one to two somatic complaints frequently. The mean value of life satisfaction (*M* = 7.07) was low. All the adolescents reported low loneliness (100%). This profile had the highest percentage of risky social media users (66%), and roughly one fifth were problematic social media users (22%).

The “Poor mental health and high loneliness” profile was the only profile with adolescents who reported high loneliness (99%) in Sample 2. This profile had the highest percentage of adolescents reporting three to four frequent psychological (59%) or somatic complaints (25%). The mean value of life satisfaction (*M* = 5.74) was the lowest in this profile. The majority were risky (52%) or problematic (20%) social media users.

### Socio-demographic description of mental health profiles

Tables 6, 7 present descriptive results from the Chi-square test and post hoc ANOVA, showing the characteristics of adolescents in each profile. The “Good mental health” profile in both samples contained more boys than girls, whereas the other three profiles contained more girls than boys. The “Somatically challenged” profile in Sample 1, and the “Poor mental health and low loneliness” and “Mixed psychosomatic health” profiles in Sample 2 had a higher proportion of adolescents whose language of instruction was Swedish than the “Good mental health” profiles. The “Poor mental health” profile in Sample 1 and the “Poor mental health and high loneliness” profile in Sample 2 had a higher proportion of first-generation immigrants and adolescents living in a single-parent family or a stepfamily than the “Good mental health” profiles.

**Table 6:** Differences between profiles in terms of socio-demographic, psychosocial, and other health-related factors in Sample 1 (2018)

	All	Profile 1	Profile 2	Profile 3	Profile 4	Profile comparison		
	*n* = 974–3149	“Good mental health” (*n* = 386–1375)	“Mixed psychosocial health” (*n* = 195–628)	“Somatically challenged” (*n* = 148–471)	“Poor mental health” (*n* = 245–675)	Overall	*p*-value	Pairwise comparison
	%/*M* (*SD*)	%/*M* (*SD*)	%/*M* (*SD*)	%/*M* (*SD*)	%/*M* (*SD*)	χ^2^/*F*		
Sample 1 (2018)								
Socio-demographic characteristics								
Gender, female (vs. male)	51.2	39.7	56.1	58.6	65.1	140.79	< 0.001	1 and 2^c^, 1 and 3^c^, 1 and 4^c^, 2 and 4^d^ differ
Grade						48.46	< 0.001	
5th	30.4	35.9	30.4	25.1	23.0			1 and 3^c^, 1 and 4^c^, 2 and 4^e^ differ
7th	36.5	34.4	36.8	41.0	37.5			No differences
9th	33.1	29.7	32.8	34.0	39.6			1 and 4^c^ differ
Language of instruction, Swedish (vs. Finnish)	19.4	16.9	21.5	24.2	19.3	14.00	0.003	1 and 3^c^ differ
Relative family affluence						20.83	0.002	
Low	17.1	15.6	19.1	18.3	17.8			No differences
Medium	57.0	60.9	55.4	50.5	54.8			1 and 3^c^, 1 and 4^e^ differ
High	25.9	23.5	25.5	31.2	27.4			1 and 3^d^ differ
Family structure						36.14	< 0.001	
Nuclear family	74.7	79.2	73.5	73.4	67.2			1 and 2^e^, 1 and 4^c^ differ
Single-parent family	13.4	11.0	14.8	13.2	17.2			1 and 4^c^ differ
Step-family	11.9	9.7	11.7	13.4	15.6			1 and 4^c^ differ
Urban residence (vs. rural)	54.9	59.4	50.7	51.7	51.9	19.93	< 0.001	1 and 2^d^, 1 and 3^e^, 1 and 4^d^ differ
Immigrant background						18.62	0.005	
First-generation immigrant	4.3	3.3	3.1	4.7	7.0			1 and 4^c^, 2 and 4^e^ differ
Second-generation immigrant	7.1	7.1	6.5	7.5	7.2			No differences
Native (non-immigrant)	88.7	89.6	90.5	87.7	85.8			No differences
Psychosocial factors								
Perceived home atmosphere	4.32 (0.78)	4.55 (0.59)	4.23 (0.73)	4.40 (0.67)	3.90 (0.99)	122.94	< 0.001	All profiles differ ^**c**^
Parental monitoring^a^								
Maternal monitoring	2.44 (0.45)	2.53 (0.40)	2.41 (0.44)	2.48 (0.42)	2.28 (0.50)	36.07	< 0.001	1 and 2^c^, 1 and 4^c^, 2 and 4^c^, 3 and 4^c^ differ
Paternal monitoring	2.24 (0.53)	2.36 (0.50)	2.23 (0.52)	2.24 (0.51)	2.04 (0.56)	37.07	< 0.001	1 and 2^c^, 1 and 3^d^, 1 and 4^c^, 2 and 4^c^, 3 and 4^c^ differ
Family support	5.70 (1.63)	6.05 (1.53)	5.57 (1.52)	5.89 (1.40)	4.99 (1.83)	71.63	< 0.001	1 and 2^c^, 1 and 4^c^, 2 and 3^d^, 2 and 4^c^, 3 and 4^c^ differ
Peer support	5.47 (1.64)	5.63 (1.55)	5.23 (1.67)	5.78 (1.40)	5.14 (1.85)	24.20	< 0.001	1 and 2^c^, 1 and 4^c^, 2 and 3^c^, 3 and 4^c^ differ
Teacher support	3.85 (0.93)	4.11 (0.77)	3.81 (0.89)	3.85 (0.88)	3.37 (1.10)	105.28	< 0.001	1 and 2^c^, 1 and 3^c^, 1 and 4^c^, 2 and 4^c^, 3 and 4^c^ differ
Classmate support	3.90 (0.77)	4.09 (0.65)	3.79 (0.76)	3.87 (0.71)	3.62 (0.92)	66.89	< 0.001	1 and 4^c^, 1 and 2^c^, 1 and 3^c^, 2 and 4^c^, 3 and 4^c^ differ
Perceived school climate	4.15 (0.86)	4.43 (0.67)	4.04 (0.85)	4.19 (0.71)	3.66 (1.06)	140.30	< 0.001	1 and 2^c^, 1 and 3^c^, 1 and 4^c^, 2 and 3^e^, 2 and 4^c^, 3 and 4^c^ differ
Intensity of online communication	3.02 (0.85)	2.92 (0.79)	2.97 (0.85)	3.08 (0.80)	3.22 (0.96)	19.64	< 0.001	1 and 2^d^, 1 and 4^c^, 2 and 4^c^, 3 and 4^e^ differ
Other health-related factors								
Academic educational expectations^b^ (vs. vocational)	67.2	65.5	75.4	71.6	60.8	12.26	0.007	2 and 4^d^ differ
Health literacy^a^						96.91	< 0.001	
Low	9.2	5.7	8.0	7.1	17.6			1 and 4^c^, 2 and 4^c^, 3 and 4^c^ differ
Moderate	56.2	52.0	64.4	55.1	57.1			1 and 2^c^ differ
High	34.6	42.3	27.6	37.8	25.3			1 and 2^c^, 1 and 4^c^, 2 and 3^e^, 3 and 4^d^ differ
Self-rated health						412.48	< 0.001	
Poor	2.2	0.2	1.1	1.3	7.9			1 and 2^e^, 1 and 3^e^, 1 and 4^c^, 2 and 4^c^, 3 and 4^c^ differ
Fair	14.0	6.4	17.0	12.3	27.9			1 and 2^c^, 1 and 3^c^, 1 and 4^c^, 2 and 4^c^, 3 and 4^c^ differ
Good	60.5	59.8	66.0	68.2	51.5			1 and 3^d^, 1 and 4^d^, 2 and 4^c^, 3 and 4^c^ differ
Excellent	23.3	33.5	15.9	18.3	12.8			1 and 2^c^, 1 and 3^c^, 1 and 4^c^ differ

Chi-square test for percentage comparison and Post-hoc ANOVA for mean comparison (two-tailed)

^a^Answered only by 7th and 9th grade adolescents (*n* = 2191)

^b^Answered only by 9th grade adolescents (*n* = 1041)

^c^Profiles differed significantly (*p* < 0.001) when Bonferroni-corrected pairwise comparisons were used

^d^Profiles differed significantly (*p* < 0.01) when Bonferroni-corrected pairwise comparisons were used

^e^Profiles differed significantly (*p* < 0.05) when Bonferroni-corrected pairwise comparisons were used

**Table 7:** Differences between profiles in terms of socio-demographic, psychosocial, and other health-related factors in Sample 2 (2022)

	All	Profile 1	Profile 2	Profile 3	Profile 4	Profile comparison		
	*n* = 738–2981	“Good mental health” (*n* = 232–1103)	“Mixed psychosomatic health” (*n* = 124–499)	“Poor mental health and low loneliness” (*n* = 254–1011)	“Poor mental health and high loneliness” (*n* = 128–368)	Overall	*p*-value	Pairwise comparison
	%/*M* (*SD*)	%/*M* (*SD*)	%/*M* (*SD*)	%/*M* (*SD*)	%/*M* (*SD*)	χ^2^/*F*		
Sample 2 (2022)								
Socio-demographic characteristics								
Gender, female (vs. male)	54.7	39.6	51.6	65.3	75.3	209.56	< 0.001	1 and 2^c^, 1 and 3^c^, 1 and 4^c^, 2 and 3^c^, 2 and 4^c^, 3 and 4^d^ differ
Grade						85.70	< 0.001	
5th	36.7	44.3	40.5	32.0	21.2			1 and 3^c^, 1 and 4^c^, 2 and 3^d^, 2 and 4^c^, 3 and 4^d^ differ
7th	36.2	33.2	32.5	39.7	40.5			1 and 3^e^, 2 and 3^e^ differ
9th	27.2	22.5	27.1	28.3	38.3			1 and 3^e^, 1 and 4^c^, 2 and 4^d^, 3 and 4^d^ differ
Language of instruction, Swedish (vs. Finnish)	35.3	30.3	39.7	40.4	30.2	31.94	< 0.001	1 and 2^d^, 1 and 3^c^, 2 and 4^e^, 3 and 4^d^ differ
Relative family affluence						22.76	< 0.001	
Low	16.8	17.8	14.0	14.8	23.4			2 and 4^d^, 3 and 4^d^ differ
Medium	56.4	57.4	59.1	55.4	52.2			No differences
High	26.8	24.8	26.9	29.8	24.5			No differences
Family structure						40.23	< 0.001	
Nuclear family	69.6	73.9	72.8	67.4	58.2			1 and 3^d^, 1 and 4^c^, 2 and 4^c^, 3 and 4^e^ differ
Single-parent family	20.4	18.8	15.8	21.8	27.7			1 and 4^d^, 2 and 3^e^, 2 and 4^c^ differ
Step-family	10.0	7.3	11.4	10.8	14.0			1 and 3^e^, 1 and 4^d^ differ
Urban residence (vs. rural)	57.2	57.8	53.1	57.7	59.8	4.62	0.202	No differences
Immigrant background						37.01	< 0.001	
First-generation immigrant	3.4	3.0	3.7	2.5	6.5			1 and 4^e^, 3 and 4^d^ differ
Second-generation immigrant	6.3	6.0	3.9	6.1	11.6			1 and 4^d^, 2 and 4^c^, 3 and 4^d^ differ
Native (non-immigrant)	90.3	91.1	92.4	91.5	81.9			1 and 4^c^, 2 and 4^c^, 3 and 4^c^ differ
Psychosocial factors								
Perceived home atmosphere	4.28 (0.80)	4.57 (0.59)	4.45 (0.65)	4.11 (0.80)	3.69 (1.05)	156.82	< 0.001	1 and 2^**e**^, 1 and 3^**c**^, 1 and 4^**c**^, 2 and 3^**c**^, 2 and 4^**c**^, 3 and 4 ^**c**^ differ
Parental monitoring^a^								
Maternal monitoring	2.49 (0.44)	2.59 (0.39)	2.56 (0.40)	2.39 (0.44)	2.31 (0.51)	63.09	< 0.001	1 and 3^**c**^, 1 and 4^**c**^, 2 and 3^**c**^, 2 and 4^**c**^, 3 and 4^**e**^ differ
Paternal monitoring	2.30 (0.55)	2.45 (0.50)	2.41 (0.49)	2.18 (0.54)	1.99 (0.62)	86.44	< 0.001	1 and 3^**c**^, 1 and 4^**c**^, 2 and 3^**c**^, 2 and 4^**c**^, 3 and 4^**c**^ differ
Family support	5.54 (1.64)	6.07 (1.40)	5.80 (1.50)	5.31 (1.56)	4.24 (1.83)	144.41	< 0.001	1 and 2^**d**^, 1 and 3^**c**^, 1 and 4^**c**^, 2 and 3^**c**^, 2 and 4^**c**^, 3 and 4^**c**^ differ
Peer support	5.42 (1.63)	5.70 (1.47)	5.54 (1.56)	5.43 (1.55)	4.36 (1.95)	68.01	< 0.001	1 and 3^**c**^, 1 and 4^**c**^, 2 and 4^**c**^, 3 and 4^**c**^ differ
Teacher support	3.93 (0.92)	4.24 (0.75)	3.96 (0.92)	3.81 (0.86)	3.26 (1.11)	124.80	< 0.001	1 and 2^**c**^, 1 and 3^**c**^, 1 and 4^**c**^, 2 and 3^**e**^, 2 and 4^**c**^, 3 and 4^**c**^ differ
Classmate support	3.76 (0.84)	4.02 (0.70)	3.88 (0.74)	3.64 (0.85)	3.14 (0.97)	121.72	< 0.001	1 and 2^**d**^, 1 and 3^**c**^, 1 and 4, 2 and 3^**c**^, 2 and 4^**c**^, 3 and 4^**c**^ differ
Perceived school climate	3.92 (0.94)	4.27 (0.74)	4.05 (0.83)	3.77 (0.91)	3.10 (1.13)	179.77	< 0.001	1 and 2^**c**^, 1 and 3^**c**^, 1 and 4^**c**^, 2 and 3^**c**^, 2 and 4^**c**^, 3 and 4^**c**^ differ
Intensity of online communication	3.03 (0.94)	2.93 (0.94)	3.04 (0.90)	3.14 (0.93)	2.98 (1.03)	8.31	< 0.001	1 and 3^**c**^ differ
Other health-related factors								
Academic educational expectations^b^ (vs. vocational)	62.9	69.0	62.9	61.0	55.5	7.07	0.070	No differences
Health literacy^a^						56.54	< 0.001	
Low	8.0	4.2	4.0	9.1	17.3			1 and 3^d^, 1 and 4^c^, 2 and 3^e^, 2 and 4^c^, 3 and 4^d^ differ
Moderate	62.2	63.4	62.0	63.0	58.1			No differences
High	29.8	32.4	34.0	27.9	24.6			No differences
Self-rated health						355.37	< 0.001	
Poor	1.9	0.4	0.8	2.3	7.1			1 and 3^d^, 1 and 4^c^, 2 and 4^c^, 3 and 4^c^ differ
Fair	13.9	5.2	8.4	19.8	31.3			1 and 3^c^, 1 and 4^c^, 2 and 3^c^, 2 and 4^c^, 3 and 4^c^ differ
Good	59.7	59.1	65.7	60.7	50.8			1 and 4^e^, 2 and 4^c^, 3 and 4^d^ differ
Excellent	24.5	35.4	25.1	17.2	10.9			1 and 2^c^, 1 and 3^c^, 1 and 4^c^, 2 and 3^d^, 2 and 4^c^, 3 and 4^e^ differ

Chi-square test for percentage comparison and Post-hoc ANOVA for mean comparison (two-tailed)

^a^Answered only by 7th and 9th grade adolescents (*n* = 1888)

^b^Answered only by 9th grade adolescents (*n* = 810)

^c^Profiles differed significantly (*p* < 0.001) when Bonferroni-corrected pairwise comparisons were used

^d^Profiles differed significantly (*p* < 0.01) when Bonferroni-corrected pairwise comparisons were used

^e^Profiles differed significantly (*p* < 0.05) when Bonferroni-corrected pairwise comparisons were used

### Factors associated with mental health profiles

Table 8 presents the results from the mixed effect multinomial logistic regression analysis, showing associations between socio-demographic characteristics, psychosocial factors, educational expectations, health literacy, self-rated health, and mental health profiles, showing the “Good mental health” profile as the reference category in both samples.

**Table 8:** Crude and adjusted OR between key variables and mental health profiles in Sample 1 (2018) and Sample 2 (2022)

Variable		Sample 1 (2018) Profile (reference: profile 1—“Good mental health”)						Sample 2 (2022) Profile (reference: profile 1— “Good mental health”)					
		Profile 2—“Mixed psychosocial health”		Profile 3—“Somatically challenged”		Profile 4—“Poor mental health”		Profile 2—“Mixed psychosomatic health”		Profile 3—“Poor mental health and low loneliness”		Profile 4—“Poor mental health and high loneliness”	
		OR (*CI* 95%)	*p*-value	OR (*CI* 95%)	*p*-value	OR (*CI* 95%)	*p*-value	OR (*CI* 95%)	*p*-value	OR (*CI* 95%)	*p*-value	OR (*CI* 95%)	*p*-value
Socio-demographic characteristics													
Gender, female (ref. male)	Crude	**1.95 (1.61–2.36)**	** < 0.001**	**2.16 (1.74–2.67)**	** < 0.001**	**2.84 (2.34–3.45)**	** < 0.001**	**1.64 (1.32–2.04)**	** < 0.001**	**2.93 (2.44–3.51)**	** < 0.001**	**4.49 (3.42–5.89)**	** < 0.001**
	Adjusted^a^	**1.94 (1.59–2.36)**	** < 0.001**	**2.11 (1.69–2.63)**	** < 0.001**	**2.94 (2.40–3.57)**	** < 0.001**	**1.61 (1.28–2.02)**	** < 0.001**	**3.11 (2.56–3.77)**	** < 0.001**	**5.69 (4.18–7.76)**	** < 0.001**
	Adjusted^b^	**1.81 (1.45–2.27)**	** < 0.001**	**1.82 (1.42–2.33)**	** < 0.001**	**2.53 (1.98–3.25)**	** < 0.001**	**1.46 (1.13–1.90)**	**0.004**	**2.49 (1.98–3.14)**	** < 0.001**	**4.87 (3.25–7.28)**	** < 0.001**
Grade (ref. 5)													
7th	Crude	1.22 (0.96- 1.54)	0.108	**1.71 (1.32–2.22)**	** < 0.001**	**1.70 (1.33–2.16)**	** < 0.001**	1.08 (0.82–1.41)	0.600	**1.56 (1.24–1.97)**	** < 0.001**	**2.69 (1.91–3.79)**	** < 0.001**
	Adjusted^a^	1.21 (0.95–1.55)	0.127	**1.67 (1.27–2.21)**	** < 0.001**	**1.58 (1.22–2.04)**	** < 0.001**	1.20 (0.90–1.60)	0.206	**1.55 (1.21–1.99)**	** < 0.001**	**2.86 (1.97–4.13)**	** < 0.001**
	Adjusted^b^	0.89 (0.68–1.18)	0.428	**1.39 (1.02–1.89)**	**0.036**	0.84 (0.61–1.14)	0.254	1.06 (0.77–1.46)	0.730	1.18 (0.89–1.57)	0.246	**1.79 (1.11–2.90)**	**0.017**
9th	Crude	**1.28 (1.01–1.63)**	**0.044**	**1.64 (1.25–2.15)**	** < 0.001**	**2.08 (1.63–2.64)**	** < 0.001**	1.32 (0.99–1.78)	0.062	**1.62 (1.25–2.09)**	** < 0.001**	3.65 (2.55–5.21)	** < 0.001**
	Adjusted^a^	**1.30 (1.01–1.67)**	**0.040**	**1.63 (1.22–2.16)**	** < 0.001**	**2.02 (1.56–2.60)**	** < 0.001**	1.35 (0.99–1.83)	0.056	**1.58 (1.20–2.07)**	** < 0.001**	**3.56 (2.42–5.23)**	** < 0.001**
	Adjusted^b^	0.90 (0.68–1.20)	0.470	1.28 (0.93–1.76)	0.130	0.91 (0.67–1.23)	0.530	0.99 (0.70–1.40)	0.968	0.99 (0.73–1.34)	0.944	**1.81 (1.10–2.98)**	**0.020**
Language of instruction, Swedish (ref. Finnish)	Crude	1.30 (0.99–1.69)	0.056	**1.57 (1.21–2.02)**	** < 0.001**	1.10 (0.84–1.43)	0.495	**1.52 (1.17–1.95)**	** < 0.001**	**1.40 (1.11–1.77)**	**0.005**	1.02 (0.71–1.47)	0.914
	Adjusted^a^	1.21 (0.90–1.63)	0.197	**1.45 (1.09–1.93)**	**0.011**	0.98 (0.73–1.32)	0.904	**1.48 (1.11–1.96)**	**0.007**	**1.63 (1.27–2.11)**	** < 0.001**	1.12 (0.78–1.60)	0.540
	Adjusted^b^	1.17 (0.84–1.61)	0.356	**1.46 (1.06–2.01)**	**0.019**	0.91 (0.65–1.29)	0.611	**1.73 (1.27–2.35)**	** < 0.001**	**1.83 (1.39–2.41)**	** < 0.001**	1.20 (0.75–1.94)	0.447
Relative family affluence (ref. low)													
Medium	Crude	**0.74 (0.57–0.95)**	**0.020**	**0.71 (0.53–0.94)**	**0.019**	0.79 (0.61–1.02)	0.067	1.26 (0.93–1.72)	0.142	1.11 (0.87–1.43)	0.390	**0.67 (0.49–0.91)**	**0.011**
	Adjusted^a^	**0.73 (0.55–0.96)**	**0.025**	**0.65 (0.48–0.89)**	**0.007**	0.88 (0.66–1.17)	0.370	1.26 (0.90–1.77)	0.180	1.15 (0.87–1.52)	0.335	0.92 (0.63–1.34)	0.680
	Adjusted^b^	0.84 (0.62–1.13)	0.249	**0.69 (0.50–0.96)**	**0.027**	0.93 (0.67–1.30)	0.672	1.41 (0.95–2.08)	0.088	1.19 (0.86–1.65)	0.297	1.57 (0.94–2.64)	0.085
High	Crude	0.88 (0.65–1.18)	0.394	1.13 (0.82–1.55)	0.448	1.02 (0.76–1.36)	0.899	1.35 (0.95–1.91)	0.093	**1.35 (1.02–1.78)**	**0.033**	0.72 (0.50–1.03)	0.071
	Adjusted^a^	0.84 (0.61–1.16)	0.285	0.99 (0.71–1.40)	0.971	1.13 (0.82–1.57)	0.457	1.22 (0.83–1.79)	0.306	1.33 (0.97–1.81)	0.075	0.91 (0.60–1.40)	0.683
	Adjusted^b^	0.97 (0.69–1.37)	0.865	0.97 (0.67–1.41)	0.889	1.17 (0.80–1.71)	0.419	1.31 (0.85–2.03)	0.218	1.23 (0.85–1.77)	0.277	1.34 (0.75–2.39)	0.317
Family structure (ref. nuclear family)													
Single-parent family	Crude	**1.44 (1.09–1.92)**	**0.011**	1.30 (0.94–1.79)	0.113	**1.82 (1.39–2.39)**	** < 0.001**	0.87 (0.64–1.18)	0.362	**1.38 (1.09–1.75)**	**0.007**	**1.85 (1.35–2.53)**	** < 0.001**
	Adjusted^a^	1.35 (1.00–1.83)	0.050	1.23 (0.88–1.73)	0.224	**1.78 (1.33–2.38)**	** < 0.001**	0.99 (0.72–1.37)	0.947	**1.50 (1.16–1.93)**	**0.002**	**1.70 (1.21–2.40)**	**0.002**
	Adjusted^b^	1.20 (0.86–1.67)	0.286	1.10 (0.76–1.60)	0.609	1.34 (0.95–1.90)	0.097	0.91 (0.63–1.32)	0.623	**1.40 (1.04–1.89)**	**0.028**	1.46 (0.94–2.28)	0.096
Step-family	Crude	1.30 (0.96–1.77)	0.096	**1.50 (1.08–2.08)**	**0.015**	**1.90 (1.43–2.52)**	** < 0.001**	**1.60 (1.09–2.34)**	**0.015**	**1.71 (1.23–2.37)**	** < 0.001**	**2.54 (1.68–3.83)**	** < 0.001**
	Adjusted^a^	1.33 (0.97–1.82)	0.080	**1.42 (1.01–1.99)**	**0.045**	**1.87 (1.39–2.51)**	** < 0.001**	**1.71 (1.16–2.52)**	**0.007**	**1.79 (1.27–2.51)**	** < 0.001**	**2.47 (1.59–3.83)**	** < 0.001**
	Adjusted^b^	1.19 (0.85–1.66)	0.323	1.40 (0.98–2.00)	0.066	1.40 (0.99–1.99)	0.056	1.39 (0.90–2.13)	0.136	1.27 (0.86–1.87)	0.238	1.50 (0.87–2.59)	0.149
Urban residence (ref. rural)	Crude	**0.71 (0.58–0.87)**	** < 0.001**	**0.73 (0.59–0.91)**	**0.004**	**0.75 (0.62–0.91)**	**0.004**	0.81 (0.64–1.03)	0.085	0.99 (0.81–1.21)	0.923	1.14 (0.86–1.53)	0.361
	Adjusted^a^	**0.73 (0.59–0.91)**	**0.004**	0.84 (0.67–1.06)	0.148	**0.72 (0.58–0.89)**	**0.003**	0.88 (0.68–1.13)	0.318	1.03 (0.83–1.29)	0.770	0.96 (0.71–1.30)	0.796
	Adjusted^b^	**0.74 (0.59–0.94)**	**0.012**	0.86 (0.67–1-11)	0.253	**0.74 (0.58–0.95)**	**0.019**	0.94 (0.72–1.24)	0.678	1.15 (0.90–1.47)	0.270	1.03 (0.70–1.54)	0.868
Immigrant background (ref. native)													
First-generation immigrant	Crude	0.91 (0.53–1.57)	0.730	1.45 (0.86–2.44)	0.167	**2.19 (1.44–3.34)**	** < 0.001**	1.19 (0.66–2.15)	0.563	0.79 (0.46–1.35)	0.385	**2.47 (1.40–4.35)**	**0.002**
	Adjusted^a^	0.92 (0.52–1.63)	0.782	1.26 (0.72–2.21)	0.423	1.56 (0.95–2.55)	0.079	1.21 (0.64–2.29)	0.550	0.80 (0.44–1.46)	0.475	1.89 (0.94–3.78)	0.072
	Adjusted^b^	0.86 (0.46–1.59)	0.620	0.80 (0.40–1.58)	0.513	1.36 (0.75–2.45)	0.314	1.15 (0.57–2.32)	0.696	0.66 (0.33–1.32)	0.237	1.26 (0.51–3.14)	0.615
Second-generation immigrant	Crude	0.91 (0.62–1.33)	0.614	1.09 (0.73–1.63)	0.675	1.07 (0.74–1.53)	0.732	0.65 (0.38–1.11)	0.115	1.01 (0.69–1.46)	0.972	**2.03 (1.32–3.12)**	**0.001**
	Adjusted^a^	0.87 (0.58–1.31)	0.510	1.14 (0.75–1.72)	0.545	1.03 (0.70–1.52)	0.883	0.68 (0.39–1.19)	0.175	1.00 (0.66–1.51)	0.993	**1.88 (1.14–3.09)**	**0.013**
	Adjusted^b^	0.77 (0.50–1.19)	0.236	1.11 (0.71–1.74)	0.648	0.75 (0.47–1.20)	0.228	**0.49 (0.26–0.94)**	**0.033**	0.85 (0.53–1.36)	0.497	1.10 (0.57–2.09)	0.784
Psychosocial factors													
Perceived home atmosphere	Crude	**0.50 (0.44–0.58)**	** < 0.001**	**0.69 (0.58–0.81)**	** < 0.001**	**0.32 (0.28–0.37)**	** < 0.001**	**0.74 (0.63–0.88)**	** < 0.001**	**0.40 (0.35–0.46)**	** < 0.001**	**0.24 (0.20–0.28)**	** < 0.001**
	Adjusted^a^	**0.51 (0.44–0.59)**	** < 0.001**	**0.76 (0.64–0.90)**	**0.002**	**0.36 (0.31–0.42)**	** < 0.001**	**0.76 (0.64–0.92)**	**0.004**	**0.42 (0.36–0.49)**	** < 0.001**	**0.26 (0.22–0.32)**	** < 0.001**
	Adjusted^b^	**0.64 (0.54–0.77)**	** < 0.001**	0.98 (0.80–1.20)	0.833	**0.56 (0.47–0.67)**	** < 0.001**	0.99 (0.79–1.25)	0.955	**0.60 (0.49–0.72)**	** <0.001**	**0.60 (0.46–0.77)**	** < 0.001**
Parental monitoring													
Maternal monitoring	Crude^1^	**0.52 (0.39–0.68)**	** < 0.001**	0.76 (0.57–1.03)	0.078	**0.29 (0.23–0.37)**	** < 0.001**	0.78 (0.56–1.10)	0.154	**0.40 (0.30–0.52)**	** < 0.001**	**0.27 (0.20–0.38)**	** <0 .001**
	Adjusted^a^^1^	**0.40 (0.30–0.54)**	** < 0.001**	**0.60 (0.44–0.83)**	**0.002**	**0.21 (0.16–0.28)**	** < 0.001**	**0.65 (0.45–0.94)**	**0.021**	**0.30 (0.22–0.40)**	** < 0.001**	**0.17 (0.11–0.24)**	** < 0.001**
	Adjusted^c^^1^	**0.41 (0.26–0.64)**	** < 0.001**	0.87 (0.54–1.41)	0.580	**0.44 (0.28–0.69)**	** < 0.001**	0.71 (0.42–1.20)	0.200	**0.44 (0.28–0.69)**	** <0.001**	0.63 (0.34–1.17)	0.144
Paternal monitoring	Crude^1^	**0.60 (0.48–0.75)**	** < 0.001**	**0.63 (0.49–0.81)**	** < 0.001**	**0.33 (0.26–0.41)**	** < 0.001**	0.86 (0.65–1.14)	0.289	**0.46 (0.37–0.57)**	** < 0.001**	**0.24 (0.18–0.32)**	** < 0.001**
	Adjusted^a^^1^	**0.60 (0.46–0.76)**	** < 0.001**	**0.65 (0.50–0.85)**	**0.001**	**0.34 (0.26–0.43)**	** < 0.001**	0.80 (0.59–1.09)	0.154	**0.45 (0.35–0.58)**	** < 0.001**	**0.24 (0.17–0.33)**	** < 0.001**
	Adjusted^c^^1^	1.38 (0.94–2.04)	0.103	0.81 (0.55–1.19)	0.285	1.00 (0.67–1.48)	0.997	1.06 (0.68–1.65)	0.785	0.91 (0.63–1.33)	0.639	0.75 (0.45–1.23)	0.255
Family support	Crude	**0.81 (0.76–0.86)**	** < 0.001**	**0.92 (0.86–0.99)**	**0.029**	**0.68 (0.64–0.72)**	** < 0.001**	**0.85 (0.78–0.92)**	** < 0.001**	**0.70 (0.66–0.75)**	** < 0.001**	**0.52 (0.48–0.56)**	** < 0.001**
	Adjusted^a^	**0.82 (0.77–0.88)**	** < 0.001**	0.95 (0.88–1.03)	0.240	**0.71 (0.67–0.75)**	** < 0.001**	**0.84 (0.77–0.91)**	** < 0.001**	**0.72 (0.67–0.77)**	** < 0.001**	**0.53 (0.49–0.58)**	** < 0.001**
	Adjusted^b^	0.97 (0.89–1.07)	0.549	0.94 (0.84–1.05)	0.250	**0.89 (0.80–0.98)**	**0.013**	0.92 (0.81–1.04)	0.183	**0.85 (0.76–0.94)**	**0.003**	**0.76 (0.66–0.87)**	** < 0.001**
Peer support	Crude	**0.86 (0.81–0.91)**	** < 0.001**	1.07 (0.99–1.15)	0.070	**0.84 (0.79–0.88)**	** < 0.001**	0.94 (0.87–1.01)	0.080	**0.89 (0.84–0.95)**	** < 0.001**	**0.64 (0.60–0.69)**	** < 0.001**
	Adjusted^a^	**0.82 (0.77–0.87)**	** < 0.001**	1.02 (0.95–1.10)	0.587	**0.78 (0.74–0.83)**	** < 0.001**	**0.88 (0.82–0.96)**	**0.002**	**0.85 (0.80–0.91)**	** < 0.001**	**0.61 (0.56–0.66)**	** < 0.001**
	Adjusted^b^	**0.90 (0.82–0.98)**	**0.019**	**1.14 (1.02–1.27)**	**0.017**	0.96 (0.87–1.05)	0.333	0.94 (0.84–1.06)	0.321	1.01 (0.92–1.12)	0.783	**0.83 (0.73–0.94)**	**0.004**
Teacher support	Crude	**0.66 (0.59–0.74)**	** < 0.001**	**0.69 (0.61–0.78)**	** < 0.001**	**0.41 (0.37–0.46)**	** < 0.001**	**0.64 (0.56–0.74)**	** < 0.001**	**0.54 (0.48–0.60)**	** < 0.001**	**0.31 (0.27–0.36)**	** < 0.001**
	Adjusted^a^	**0.68 (0.60–0.77)**	** < 0.001**	**0.73 (0.64–0.83)**	** < 0.001**	**0.45 (0.40–0.50)**	** < 0.001**	**0.62 (0.54–0.71)**	** < 0.001**	**0.56 (0.49–0.63)**	** < 0.001**	**0.33 (0.28–0.39)**	** < 0.001**
	Adjusted^b^	0.94 (0.81–1.09)	0.420	0.89 (0.76–1.05)	0.169	**0.73 (0.63–0.85)**	** < 0.001**	**0.65 (0.53–0.79)**	** < 0.001**	**0.80 (0.67–0.95)**	**0.011**	**0.65 (0.51–0.82)**	** < 0.001**
Classmate support	Crude	**0.56 (0.49–0.63)**	** < 0.001**	**0.64 (0.55–0.74)**	** < 0.001**	**0.43 (0.38–0.49)**	** < 0.001**	**0.77 (0.67–0.90)**	** < 0.001**	**0.52 (0.46–0.59)**	** < 0.001**	**0.28 (0.24–0.33)**	** < 0.001**
	Adjusted^a^	**0.59 (0.51–0.68)**	** < 0.001**	**0.69 (0.59–0.80)**	** < 0.001**	**0.50 (0.44–0.58)**	** < 0.001**	**0.79 (0.68–0.93)**	**0.004**	**0.55 (0.49–0.63)**	** < 0.001**	**0.33 (0.28–0.40)**	** < 0.001**
	Adjusted^b^	0.86 (0.71–1.03)	0.091	**0.81 (0.66–0.99)**	**0.041**	0.97 (0.80–1.18)	0.783	1.10 (0.88–1.38)	0.382	0.87 (0.72–1.05)	0.155	0.80 (0.62–1.04)	0.096
Perceived school climate	Crude	**0.50 (0.44–0.57)**	** < 0.001**	**0.62 (0.54–0.72)**	** < 0.001**	**0.33 (0.29–0.37)**	** < 0.001**	**0.70 (0.61–0.81)**	** < 0.001**	**0.49 (0.43–0.54)**	** < 0.001**	**0.26 (0.22–0.30)**	** < 0.001**
	Adjusted^a^	**0.53 (0.46–0.61)**	** <0 .001**	**0.67 (0.57–0.78)**	** < 0.001**	**0.37 (0.32–0.42)**	** < 0.001**	**0.72 (0.62–0.83)**	** < 0.001**	**0.53 (0.47–0.60)**	** < 0.001**	**0.29 (0.24–0.34)**	** < 0.001**
	Adjusted^b^	**0.72 (0.60–0.85)**	** < 0.001**	0.82 (0.67–1.00)	0.052	**0.57 (0.47–0.68)**	** < 0.001**	0.90 (0.72–1.12)	0.339	**0.78 (0.65–0.95)**	**0.012**	**0.49 (0.38–0.63)**	** < 0.001**
Intensity of online communication	Crude	1.06 (0.95–1.20)	0.296	**1.26 (1.10–1.43)**	** < 0.001**	**1.52 (1.36–1.71)**	** < 0.001**	**1.14 (1.01–1.29)**	**0.032**	**1.27 (1.15–1.40)**	** < 0.001**	1.06 (0.92–1.22)	0.449
	Adjusted^a^	1.02 (0.90–1.16)	0.740	1.14 (0.99–1.31)	0.065	**1.43 (1.26–1.62)**	** < 0.001**	1.09 (0.96–1.25)	0.193	**1.21 (1.08–1.35)**	** < 0.001**	0.88 (0.74–1.04)	0.120
	Adjusted^b^	**1.21 (1.06–1.39)**	**0.006**	**1.22 (1.05–1.41)**	**0.010**	**1.85 (1.60–2.14)**	** < 0.001**	**1.22 (1.06–1.41)**	**0.007**	**1.42 (1.24–1.61)**	** < 0.001**	1.21 (0.99–1.48)	0.057
Other health-related factors													
Academic educational expectations (ref. vocational)	Crude^2^	**1.60 (1.08–2.36)**	**0.018**	1.33 (0.88–2.01)	0.182	0.81 (0.58–1.14)	0.229	0.78 (0.49–1.23)	0.282	0.70 (0.48–1.03)	0.070	**0.59 (0.37–0.93)**	**0.024**
	Adjusted^a^^2^	1.46 (0.96–2.22)	0.077	0.97 (0.62–1.52)	0.886	**0.61 (0.42–0.88)**	**0.009**	0.78 (0.48–1.29)	0.341	**0.63 (0.41–0.97)**	**0.037**	**0.57 (0.33–0.99)**	**0.047**
	Adjusted^d^^2^	**1.67 (1.02–2.73)**	**0.041**	1.24 (0.73–2.09)	0.425	1.05 (0.64–1.71)	0.854	1.09 (0.58–2.04)	0.793	0.72 (0.42–1.26)	0.250	0.63 (0.30–1.33)	0.222
Health literacy (ref. low)													
Moderate	Crude^1^	0.87 (0.55–1.38)	0.563	0.85 (0.51–1.41)	0.528	**0.36 (0.24–0.52)**	** < 0.001**	1.06 (0.52–2.15)	0.874	**0.46 (0.29–0.75)**	**0.002**	**0.23 (0.13–0.38)**	** < 0.001**
	Adjusted^a^^1^	0.86 (0.53–1.38)	0.523	0.86 (0.50–1.47)	0.582	**0.36 (0.23–0.54)**	** < 0.001**	0.97 (0.47–2.01)	0.931	**0.47 (0.28–0.81)**	**0.006**	**0.20 (0.11–0.37)**	** < 0.001**
	Adjusted^c^^1^	1.43 (0.81–2.50)	0.215	1.36 (0.71–2.60)	0.355	0.72 (0.43–1.22)	0.225	1.00 (0.37–2.66)	0.993	**0.46 (0.22–0.98)**	**0.043**	**0.28 (0.12–0.70)**	**0.006**
High	Crude^1^	**0.46 (0.29–0.75)**	**0.002**	0.72 (0.43–1.20)	0.206	**0.19 (0.13–0.29)**	** < 0.001**	1.15 (0.56–2.40)	0.700	**0.42 (0.26–0.70)**	** < 0.001**	**0.18 (0.10–0.32)**	** < 0.001**
	Adjusted^a^^1^	**0.46 (0.27–0.76)**	**0.002**	0.69 (0.40–1.21)	0.193	**0.18 (0.11–0.28)**	** < 0.001**	0.99 (0.47–2.11)	0.984	**0.41 (0.23–0.72)**	**0.002**	**0.15 (0.08–0.29)**	** < 0.001**
	Adjusted^c^^1^	1.10 (0.60–2.02)	0.759	1.34 (0.67–2.68)	0.400	0.59 (0.33–1.05)	0.071	1.00 (0.37–2.66)	0.489	0.65 (0.29–1.43)	0.282	0.46 (0.17–1.20)	0.113
Self-rated health (ref. poor)													
Fair	Crude	0.52 (0.13–2.05)	0.347	0.32 (0.08–1.35)	0.122	**0.12 (0.04–0.40)**	** < 0.001**	0.73 (0.17–3.08)	0.663	0.65 (0.22–1.97)	0.450	**0.32 (0.11–0.98)**	**0.046**
	Adjusted^a^	0.53 (0.13–2.14)	0.372	0.36 (0.09–1.52)	0.164	**0.15 (0.04–0.50)**	**0.002**	1.04 (0.22–4.99)	0.964	0.79 (0.25–2.52)	0.695	0.67 (0.20–2.30)	0.527
	Adjusted^b^	0.68 (0.16–2.92)	0.602	0.29 (0.07–1.25)	0.097	**0.22 (0.06–0.80)**	**0.022**	0.97 (0.13–7.47)	0.975	0.65 (0.13–3.21)	0.597	0.81 (0.14–4.68)	0.816
Good	Crude	**0.22 (0.06–0.84)**	**0.027**	**0.19 (0.05–0.78)**	**0.021**	**0.02 (0.01–0.08)**	** < 0.001**	0.50 (0.12–2.03)	0.335	**0.18 (0.06–0.53)**	**0.002**	**0.05 (0.02–0.14)**	** < 0.001**
	Adjusted^a^	**0.22 (0.06–0.87)**	**0.030**	**0.21 (0.05–0.85)**	**0.028**	**0.03 (0.01–0.10)**	** < 0.001**	0.75 (0.16–3.44)	0.715	**0.23 (0.07–0.69)**	**0.009**	**0.11 (0.03–0.36)**	** < 0.001**
	Adjusted^b^	0.36 (0.09–1.50)	0.158	**0.20 (0.05–0.82)**	**0.026**	**0.06 (0.02–0.22)**	** < 0.001**	0.94 (0.13–6.90)	0.954	0.27 (0.06–1.27)	0.097	0.25 (0.05–1.40)	0.116
Excellent	Crude	**0.09 (0.02–0.37)**	** < 0.001**	**0.09 (0.02–0.38)**	** < 0.001**	**0.01 (0.00–0.04)**	** < 0.001**	0.32 (0.08–1.31)	0.114	**0.09 (0.03–0.25)**	** < 0.001**	**0.02 (0.01–0.05)**	** < 0.001**
	Adjusted^a^	**0.10 (0.03–0.42)**	** < 0.001**	**0.11 (0.03–0.46)**	**0.002**	**0.02 (0.00–0.05)**	** < 0.001**	0.52 (0.11–2.39)	0.400	**0.12 (0.04–0.38)**	** < 0.001**	**0.04 (0.01–0.14)**	** < 0.001**
	Adjusted^b^	**0.23 (0.05–0.97)**	**0.046**	**0.12 (0.03–0.50)**	**0.004**	**0.04 (0.01–0.15)**	** < 0.001**	0.77 (0.10–5.70)	0.798	**0.18 (0.04–0.89)**	**0.036**	**0.15 (0.02–0.86)**	**0.033**

Mixed-effect multinomial logistic regression models per profile: odds ratios (OR), 95% confidence intervals (*CI*), ref. reference category. Bold values denote statistical significance

^**1**^Only 7th and 9th grade adolescents included

^**2**^Only 9th grade adolescents included

^**a**^Adjusted for socio-demographic characteristics (gender, grade level, language of instruction, relative family affluence, family structure, urban/rural residence, immigrant background)

^**b**^Adjusted for socio-demographic characteristics, psychosocial factors (perceived social support, perceived home atmosphere, intensity of online communication, perceptions of school cohesion), and self-rated health

^**c**^Adjusted for socio-demographic characteristics, psychosocial factors (perceived social support, perceived home atmosphere, intensity of online communication, perceptions of school cohesion, parental monitoring), and other health-related factors (self-rated health and health literacy)

^**d**^Adjusted for socio-demographic characteristics, psychosocial factors (perceived social support, perceived home atmosphere, intensity of online communication, perceptions of school cohesion, parental monitoring), and other health-related factors (self-rated health, health literacy, and educational expectations)

#### Sample 1 (2018)

After adjustment for all variables (model adjusted b–d), in 2018, adolescents belonging to any of the other three profiles than the “Good mental health” profile were more likely to be girls, and to report a higher intensity of online communication. In addition, they were less likely to report excellent self-rated health than poor self-rated health.

Those belonging to the “Mixed psychosocial health” profile were also more likely to report lower maternal monitoring, lower peer support, and a less positive home atmosphere and school climate, and to have academic educational expectations, and were less likely to live in an urban residence than those in the “Good mental health” profile.

Those belonging to the “Somatically challenged” profile were also more likely to be in the 7th grade, to have Swedish as opposed to Finnish as their language of instruction, and to report higher peer support and lower classmate support, and were less likely to report medium family affluence than low family affluence than those in the “Good mental health” profile.

Those belonging to the “Poor mental health” profile were more likely to report lower maternal monitoring, lower family support, lower teacher support, and a less positive home atmosphere and school climate, and were less likely to live in an urban residence than those in the “Good mental health” profile.

After adjustment for all variables, family structure, immigrant background, paternal monitoring and health literacy were not associated with profile membership (*p* > 0.05).

For the school-level variance in the models, see Additional file 1: Table S4.

#### Sample 2 (2022)

After adjustment for all variables (model adjusted b–d), in 2022, adolescents belonging to any of the other three profiles than the “Good mental health” profile were more likely to be girls, and to report lower teacher support.

Those belonging to the “Mixed psychosomatic health” profile were also more likely to have Swedish as their language of instruction, more likely to report a higher intensity of online communication, and less likely to have a second-generation immigrant background than a native background than those in the “Good mental health” profile.

Those belonging to the “Poor mental health and low loneliness” profile were more likely to have Swedish as their language of instruction; to live in a single-parent family; to report lower maternal monitoring, lower family support, higher intensity of online communication, and a less positive home atmosphere and school climate; and less likely to report moderate health literacy (reference category, ref., low health literacy) and excellent self-rated health (ref. poor self-rated health) than those in the “Good mental health” profile.

Those belonging to the “Poor mental health and high loneliness” profile were more likely to be in the 7th or 9th grade and to report lower family and peer support and a less positive home atmosphere and school climate; and were less likely to report moderate health literacy (ref. low health literacy) and excellent self-rated health (ref. poor self-rated health) than those in the “Good mental health” profile.

After adjustment for all variables, family affluence, urban residence, classmate support, paternal monitoring, and educational expectations had no relationship with profile membership (*p* > 0.05).

## Discussion

To show the diversity in adolescents’ mental health reactions to the COVID-19 pandemic, this study identified mental health profiles in two samples of Finnish adolescents before (2018) and after (2022) the peak of the pandemic, and examined how the emerging profiles were associated with a range of health-related factors. We identified four profiles in both samples, showing the advantage of a person-oriented approach when examining diverse complex manifestations of mental health among youths. The identified profiles further differed in terms of several socio-demographic, psychosocial, and other health-related factors.

In our study, nearly half (44%) of the adolescents in Sample 1 and roughly one-third (37%) of those in Sample 2 belonged to the “Good mental health” profile, were mainly normative social media users, and experienced no frequent health complaints, low loneliness, and high life satisfaction. Importantly, however, a somewhat smaller proportion of adolescents belonged to this healthier profile (i.e., “Good mental health”) in the second sample, which might indicate that adolescents’ mental health has deteriorated during the pandemic, thus supporting previous research [4]. The two timepoints also shared another somewhat similar profile (i.e., “Mixed psychosocial health” and “Mixed psychosomatic health”), which was characterized by average life satisfaction and for the most part, frequent psychological complaints, low loneliness, and normative social media use. However, in Sample 2, the majority experienced frequent somatic complaints, whereas in Sample 1, no frequent somatic complaints were reported. This finding might indicate that in 2022, comorbidity of psychological and somatic complaints was more common.

We also identified differences between the two samples. In the first sample (2018), in addition to a poor mental health profile, one exceptional profile emerged in which all adolescents experienced frequent somatic complaints, but not necessarily psychological complaints. In the second sample (2022), two profiles were characterized by poor mental health, with almost all the adolescents (99%) in one reporting high loneliness. It seems that in the second sample, adolescents’ perceived loneliness was more closely linked to their other mental health problems than in the first sample, which may indicate that lonely adolescents are especially vulnerable to the negative impact of the pandemic. This should also be acknowledged in measures to promote post-pandemic recovery, as adolescents’ loneliness has increased since the onset of the pandemic [55, 80, 81]. However, in our study, in the three profiles characterized by poorer mental health in both samples, the standard deviation of life satisfaction was greater than in the “Good mental health” profiles, suggesting more variance in how satisfied adolescents were with their lives in the poorer profiles.

In both samples, being a girl and reporting lower maternal monitoring; lower family, peer, and teacher support; higher intensity of online communication; less positive home atmosphere and school climate; having Swedish as the language of instruction (i.e., belonging to a linguistic minority group); and being older (i.e., in 7th or 9th grade) were linked to belonging to at least one of the three poorer mental health profiles, whereas those reporting excellent self-rated health were more likely to belong to the “Good mental health” profiles. In addition, in Sample 1, reporting lower classmate support, higher peer support, and low family affluence, and having academic educational expectations were linked to some of the profiles characterized by poorer mental health, whereas those living in an urban residence were more likely to belong to the “Good mental health” profile. In Sample 2, reporting low health literacy and living in a single-parent family was also associated with belonging to at least one profile of poorer mental health, whereas having a native (non-immigrant) background was associated with belonging to the “Mixed psychosomatic health” profile. Paternal monitoring was not linked to profile membership in either sample when all variables were adjusted for.

Overall, our findings showed that the psychosocial support variables and self-rated health were more strongly related to profile membership than socio-demographic characteristics. This is in line with previous research that has shown that social support and self-rated health has a stronger effect on mental well-being than demographic and socioeconomic characteristics [82]. Moreover, in our study, the key psychosocial factors were teacher support, school climate, maternal monitoring, and home atmosphere, which implies that both school context and family environment may play a key role in adolescents’ mental health. We also found that adolescents reporting poor mental health combined with high loneliness reported more severe deficits in, for example, psychosocial support.

Our result that girls were more likely than boys to belong to the profiles reporting poorer mental health is in line with that of previous research carried out before and during the pandemic, showing that mental health problems are more common among girls [1, 83, 84]. Several biological, social, economic, and political explanations for gender differences in health outcomes have been provided (see Bambra et al. [85]). For example, evidence has shown that girls experience higher pressure and demands from school than boys, and this has been strongly linked to experiencing health complaints [86]. Moreover, it has been suggested that girls are exposed to earlier sexualization and greater body objectification, which have been associated with depressive symptoms [87]. As Finland has been ranked as having high levels of gender equality [88], our findings on gender differences could also be linked to the equality paradox of health, suggesting that individuals living in countries with greater levels of gender equality report larger gender gaps in health outcomes favoring boys [89]. However, it is also possible that poor mental health among boys is manifested in other ways not measured in our study, such as increased anti-social behaviors and substance use [90, 91].

In line with our finding that lower maternal monitoring was related to the poorer mental health profiles and that paternal monitoring was not associated with profile membership, previous research has also suggested that the mother–child relationship has stronger effects on adolescents’ well-being than the father–child relationship [92]. Many parents have faced unexpected challenges during the pandemic, and several studies have observed that symptoms of stress, anxiety, and depression have increased among parents during this time period, particularly among mothers, from pre‐pandemic estimates [93–95]. This could potentially be related to gender-based parenting roles during the pandemic, with childcare responsibilities tending to fall on mothers [96]. Furthermore, Racine et al. [95] found that mothers who have had difficulty balancing children’s home schooling with working from home and other household responsibilities during the pandemic reported more depression and anxiety than those who did not experience these challenges. When mothers are stressed by pandemic-related challenges, they may show less interest in or strength to monitor their child’s activities, which could also affect the mental health of adolescents. Already prior to the pandemic, higher stress levels among parents were shown to predict poorer child outcomes, such as depression [97], and they were also longitudinally associated with more adjustment problems (e.g., emotional problems) among adolescents during the pandemic [98]. Thus, our findings also highlight that the well-being of mothers should be taken into account in measures to promote post-pandemic recovery.

Our results also indicated that partly different risk factors were associated with belonging to the poorer mental health profiles at the two timepoints. For example, in the second sample, health literacy was linked to poorer health profile membership, whereas in the first sample, this association was non-significant. Thus, the role of health literacy should be acknowledged not only during the pandemic (e.g., in terms of abilities to follow safety regulations and to seek timely help), but also in measures to promote post-pandemic recovery, as a health asset that needs to be empowered. For this reason, supporting teachers’ and other school personnel’ capacities to develop children’s and adolescents’ health literacy is essential, as school-based health education provides an excellent opportunity to facilitate equity in learning these skills through the school curriculum. It is also important to educate health professionals to communicate health information in a clear and age-appropriate manner, as individuals with low health literacy are at particular risk of misunderstanding or ignoring advice [99]. We also found that support from teachers was more strongly linked to adolescents’ mental health profiles in the second sample, suggesting that the importance of teacher support (see also Guo et al. [100]; Wright & Wachs [101]) might have increased during the pandemic.

New opportunities to interact with others have arisen as a result of the pandemic, and some previous evidence shows that positive online experiences may have buffered experiences of loneliness during this time period [102]. In our study, however, more frequent online communication was associated with belonging to poorer mental health profiles. This is in line with a previous study that observed that adolescents who reported higher depression spent more time to connect with friends virtually during the pandemic [31]. Moreover, Cauberghe et al. [25] observed that using social media for social reasons (e.g., to compensate for the missing of friends and to talk with family and friends) was associated with higher anxiety and loneliness among adolescents during COVID-19 lockdown. However, due to the cross-sectional design of these studies, we cannot conclude whether social media use prospectively affects mental health, or vice versa. Evidence from pre-pandemic longitudinal evidence on the direction of the associations has been mixed. For example, Frison and Eggermont [103] found that adolescents who browsed more often through Instagram (i.e. a social networking site) had a higher chance to develop higher depression later on and that initial depressed mood was associated with later increases in posting on Instagram. Thus, it is possible that certain types of activities on social media may lead to poorer mental health, and that also greater engagement in certain activities on social media may follow from prior mental health problems. On the other hand, other studies found no longitudinal links between initial frequency of social media use and depression [104–106]. It has also been suggested that adolescents differ in their susceptibility to the effects of social media use. For example, Beyens et al. [107] noticed that most adolescents do not experience any short-term changes in well-being related to their duration of passive social media use (e.g., viewing posts or reading messages), and if they do experience any changes, these are more likely to be positive than negative. In their study, the duration of adolescents’ active social media use (e.g., sending messages or sharing posts) did not affect their well-being. Associations between social media use and mental health outcomes may also vary depending on the reasons for using social media. In a longitudinal study, initial higher levels of using social media to connect with others or to alleviate boredom were prospectively associated with higher levels of anxiety and problematic social media use, but also higher empathy [108]. However, using social networking sites to seek information was not related to any mental health outcomes, and none of the three reasons for using social media studied was associated with depression or life satisfaction. Other evidence has shown that associations between online communication and well-being might be positive or negative depending on whom adolescents interact with online (e.g., peers, unknown people) [109].

Somewhat unexpected findings from our study were that having academic educational expectations and living in a rural residence were linked to poorer mental health in the first sample. This is contrary to previous studies which have observed that youths who have higher educational expectations [33] and live in rural areas [48, 49] tend to report better mental health. Although we do not know the direction of the associations, potential explanation for our results could be that those who have academic educational expectations may experience higher academic stress, which has been linked to poorer mental health [110], and that rural living may comprise negative experiences of social exclusion, insufficient activities, and limited access to resources, facilities, and transportation (for review, see Powell et al. [111]). However, educational expectations and rural residence were quite weakly associated with profile membership in our study, and the links were non-significant in the second sample when all variables were adjusted for. It should be noted that a significant educational reform took place in Finland during the fall of 2021, raising the age of compulsory education to 18 years and extending compulsory education to upper secondary education, which may have had an impact on adolescents’ educational expectations in the second sample. Another surprising finding in our study was that higher peer support was linked to belonging to the “Somatically challenged” profile in the first sample, which differs from previous research that observed negative associations between peer support and somatic [112] or psychosomatic complaints [113] among young people. However, lower peer support was also linked to profiles of poorer mental health in both samples in our study.

Our finding that adolescents who had Swedish as opposed to Finnish as the language of instruction were more likely to belong to some of the poor mental health profiles was also unexpected. In Finland, Swedish-speaking Finns represent a national linguistic minority, and children belonging to this community typically attend Swedish-speaking schools. Therefore, the language of instruction in school represents in their case also their minority status. However, previous research [65, 114, 115] has observed that this particular minority tend have better health and well-being compared with the national majority, i.e., Finnish-speaking Finns, although more recent evidence on adolescents’ health showed no differences in several outcomes (e.g., self-rated health) between these two language groups [116]. The health disparities have typically been explained by the more cohesive linguistic community ties of the Swedish-speaking Finns [117, 118]. Thus, the result of our study calls for more attention towards Swedish-speaking youngsters and their well-being in schools and specifically during social isolation.

The findings of our study have practical implications for public policies. First, our study stresses the importance of assessing several health outcomes, including loneliness, among youth, as they might be differentially related to risk and protective factors. Second, health-promoting programs should involve adolescents, their families, and the school environment. Our study points out that fostering positive teacher-student relationships, developing stronger health literacy skills among adolescents, promoting a positive home environment, and encouraging parents to keep track of their child’s activities are possible areas for future family- and school-based health-promoting interventions. In addition, more attention should be paid to girls and lonely adolescents, and those rating their health as poor, as these are most vulnerable to experiencing internalized mental health problems. Our study also highlights the potential mental health risks for adolescents who belong to a linguistic minority group, are older, and live in a single-parent household.

Several limitations of this study should be noted. First, our findings were based on self-reports, and several single-item measures were used (e.g., life satisfaction, loneliness). Using a single-item measure of loneliness might result in underestimated reports of loneliness, as respondents may be unwilling to identify themselves as “lonely” [119]. However, the single-item measure of loneliness used in this study has been shown to correlate strongly (*r* = 0.62) with multi-item measures [120] and may have been easier for the youngest participants to understand. Second, although the samples were drawn using the same cluster sampling method, the second sample was slightly younger and included more respondents with Swedish as their language of instruction than the first sample due to small socio-demographic differences in response rates. The slight change in the loneliness measure between the two timepoints may also have affected the results. In addition, it should be noted that the four types of social support were measured using two different scales, which might have affected how the respondents assessed the different support sources. Another limitation is that 10 percent of the respondents in Sample 1, and 22 percent of the respondents in Sample 2 were ineligible for the cluster analysis, and there were small variations in socio-demographic variables between those who were eligible for this analysis and those who were excluded. For example, in both samples, the excluded participants were more likely to be boys and first-generation immigrants compared to their included counterparts, meaning that girls and adolescents with a native background were overrepresented in the mental health profiles. Furthermore, our study was cross-sectional, which prevents establishing the causality or directions of relationships. For example, whether higher support improves mental health, or whether adolescents reporting frequent health complaints assess their social support as inadequate remains unconfirmed on the basis of these findings. More longitudinal research with long-term follow-up is needed to examine the direction of these associations. The data for the current study were collected in the spring term of 2022, and the most intense reactions to the pandemic might already have been over by then. However, as in many other countries, there was a dramatic increase in reported COVID cases in the spring of 2022 [121]. Later that spring, there was a slight decline in reported cases, but the number of patients receiving hospital care due to COVID-19 was still high [122]. Finnish adolescents might also have experienced additional stress during spring 2022 due to the Russia–Ukraine conflict, as Finland is bordered in the east by Russia. In addition, schools in some municipalities were closed for one week in May due to a teacher strike. For these reasons, it is not possible to conclude whether potential declines in mental health in our study might have been caused by the COVID-19 pandemic. It should also be noted that Finnish adolescents’ mental health has worsened already during the last two decades prior to the pandemic [57]. Further research should examine how adolescents’ mental health evolves during the progression of the pandemic. If the pandemic increased loneliness and increased avoidance of or reduced reward from social interaction for some adolescents, those being more resilient might be able to socially reengage more quickly, while others may have longer periods of loneliness lasting beyond the pandemic.

## Conclusions

Overall, our findings show the importance of social support and self-rated health for mental health outcomes among adolescents. They also highlighted how some specific factors assisted the adolescents in coping with the existential health-related threat. Namely, we found that the role of health literacy (e.g., having knowledge on health issues and the ability to seek and assess health-related information) and teacher support (e.g., perceiving teachers as caring and accepting) in mental health has increased during the pandemic, as these were key factors associated with better health profile membership in the second sample.

## Supplementary Information

**Additional file 1: Table S1.** Fisher r-to-z transformations showing differences between correlations in the samples. **Table S2.** Comparison of socio-demographic characteristics between included and excluded adolescents in cluster analysis in Sample 1 (2018) and Sample 2 (2022). **Table S3.** Adjusted OR between socio-demographic characteristics and respondents included or excluded in cluster analysis in Sample 1 (2018) and Sample 2 (2022). **Table S4.** School-level variance in mixed effect multinomial logistic regression analysis models.

## Data Availability

The datasets used and analyzed in the current study are available from the corresponding author on reasonable request.
