# Supplementary material for: Mental health profiles of Finnish adolescents before and after the peak of the COVID-19 pandemic
Source: Child Adolesc Psychiatry Ment Health. 2023 Apr 29;17:54. doi: 10.1186/s13034-023-00591-1 (PMC10148589; doi:10.1186/s13034-023-00591-1)
Supplement: Supplementary file 1 — Additional file 1: Table S1. Fisher r-to-z transformations showing differences between correlations in the samples. Table S2. Comparison of socio-demographic characteristics between included and excluded adolescents in cluster analysis in Sample 1 (2018) and Sample 2 (2022). Table S3. Adjusted OR between socio-demographic characteristics and respondents included or excluded in cluster analysis in Sample 1 (2018) and Sample 2 (2022). Table S4. School-level variance in mixed effect multinomial logistic regression analysis models. [file 13034_2023_591_MOESM1_ESM.docx]

Additional file 1

Additional file 1: **Table S1.** Fisher r-to-z transformations showing differences between correlations in the samples.

| Variables | Correlation coefficients | | *z*-score | *p*-value |
| --- | --- | --- | --- | --- |
|  | Sample 1 (2018) | Sample 2 (2022) |  |  |
| Psychological complaints x somatic complaints | .45 | .51 | -3.306 | **< .001** |
| Psychological complaints x perceived loneliness | .37 | .31 | 2.855 | **.004** |
| Psychological complaints x life satisfaction | .40 | .46 | -3.036 | **.002** |
| Psychological complaints x problematic social media use | .26 | .33 | -3.094 | **.002** |
| Somatic complaints x perceived loneliness | .22 | .25 | -1.333 | .183 |
| Somatic complaints x  life satisfaction | .26 | .33 | -3.159 | **.002** |
| Somatic complaints x  problematic social media use | .18 | .23 | -2.104 | **.035** |
| Perceived loneliness x  life satisfaction | .33 | .32 | 0.459 | .646 |
| Perceived loneliness x  problematic social media use | .17 | .19 | -0.830 | .406 |
| Life satisfaction x problematic social media use | .22 | .28 | -2.516 | **.012** |

Additional file 1: **Table S2.** Comparison of socio-demographic characteristics between included and excluded adolescents in cluster analysis in Sample 1 (2018) and Sample 2 (2022).

|  | Sample 1 (2018) | | | Sample 2 (2022) | | |
| --- | --- | --- | --- | --- | --- | --- |
|  | Included respondents,  *n* = 3082–3149 | Excluded respondents,  *n* = 274–349 | Significance | Included respondents,  *n* = 2789–2981 | Excluded respondents,  *n* = 547–857 | Significance |
|  | % (N) / M (SD) | % (N) / M (SD) |  | % (N) / M (SD) | % (N) / M (SD) |  |
| Socio-demographic characteristics |  |  |  |  |  |  |
| Gender, female (vs. male) | **51.2 (1602)** | **37.2 (124)** | **ꭙ2 = 23.57, *p* < .001** | **54.7 (1613)** | **36.3 (302)** | **ꭙ2 = 87.54, *p* < .001** |
| Mean age | **13.41 (1.69)** | **13.70 (1.71)** | **t(3476) = -2.93, *p* = .003** | 13.24 (1.73) | 13.10 (1.77) | **t(3798) = 2.06, *p* = .039** |
| Grade |  |  | **ꭙ2 = 7.70, *p* = .021** |  |  | **ꭙ2 = 11.75, *p* = .003** |
| 5^th^ | **30.4 (958)** | **23.8 (83)** | ***p* = .010 ^d^** | 36.7 (1093) | 39.6 (339) | *p* > .05 ^d^ |
| 7^th^ | 36.5 (1150) | 37.5 (131) | *p* > .05 ^d^ | 36.2 (1078) | 39.1 (335) | *p* > .05 ^d^ |
| 9^th^ | **33.1 (1041)** | **38.7 (135)** | ***p* = .035 ^d^** | **27.2 (810)** | **21.4 (183)** | ***p* = .001 ^d^** |
| Language of instruction, Swedish (vs. Finnish) | 19.4 (612) | 22.6 (79) | ꭙ2 = 2.03, *p* = .154 | **35.3 (1051)** | **43.5 (373)** | **ꭙ2 = 19.50, *p* < .001** |
| Relative family affluence |  |  | **ꭙ2 = 16.78, *p* < .001** |  |  | **ꭙ2 = 10.97, *p* = .004** |
| Low | **17.1 (540)** | **25.5 (70)** | ***p* = .001 ^d^** | **16.8 (501)** | **21.6 (179)** | ***p* = .001 ^d^** |
| Medium | **57.0 (1794)** | **45.5. (125)** | ***p* < .001 ^d^** | **56.4 (1680)** | **51.8 (428)** | ***p* = .018 ^d^** |
| High | 25.9 (815) | 29.1 (80) | *p* > .05 ^d^ | 26.8 (799) | 26.6 (220) | *p* > .05 ^d^ |
| Family structure |  |  | ꭙ2 = 3.46, *p* = .178 |  |  | ꭙ2 = 5.12, *p* = .077 |
| Nuclear family | 74.7 (2302) | 75.2 (206) | *p* > .05 ^d^ | 69.6 (1942) | 67.8 (371) | *p* > .05 ^d^ |
| Single-parent family | 13.4 (413) | 16.1 (44) | *p* > .05 ^d^ | **20.4 (568)** | **24.1 (132)** | ***p* = .048 ^d^** |
| Stepfamily | 11.9 (367) | 8.8 (24) | *p* > .05 ^d^ | 10.0 (279) | 8.0 (44) | *p* > .05 ^d^ |
| Urban residence (vs. rural) | 54.9 (1722) | 58.2 (189) | ꭙ2 = 1.27, *p* = .261 | 57.2 (1693) | 56.9 (459) | ꭙ2 = 0.03, *p* = .863 |
| Immigrant background |  |  | **ꭙ2 = 6.79, *p* = .034** |  |  | **ꭙ2 = 32.35, *p* < .001** |
| First-generation immigrant | **4.3 (133)** | **7.1 (23)** | ***p* = .019 ^d^** | **3.4 (97)** | **7.3 (57)** | ***p* < .001 ^d^** |
| Second-generation immigrant | **7.1 (219)** | **8.6 (28)** | ***p* = .021 ^d^** | **6.3 (183)** | **9.0 (70)** | ***p* = .008 ^d^** |
| Native (non-immigrant) | 88.7 (2753) | 84.3 (273) | *p* > .05 ^d^ | **90.3 (2606)** | **83.7 (650)** | ***p* < .001 ^d^** |

*Note.* Chi-square test for percentage comparison and independent t-test for mean comparison.

**^d^** Bonferroni-corrected *p*-values for multiple testing.

Additional file 1: **Table S3.** Adjusted OR between socio-demographic characteristics and respondents included or excluded in cluster analysis in Sample 1 (2018) and Sample 2 (2022).

| Variable | Sample 1 (2018), *n* = 3343 | | Sample 2 (2022), *n* = 3576 | |
| --- | --- | --- | --- | --- |
|  | OR (CI 95%) | *p*-value | OR (CI 95%) | *p*-value |
| Gender, female (ref. male) | **1.65 (1.26-2.14)** | **<.001** | **2.15 (1.82-2.56)** | **<.001** |
| Grade (ref. 5) |  |  |  |  |
| 7^th^ | 0.91 (0.65-1.29) | .600 | 0.97 (0.75-1.24) | .785 |
| 9^th^ | 0.89 (0.63-1.25) | .495 | **1.43 (1.08-1.90)** | **.012** |
| Language of instruction, Swedish (ref. Finnish) | - | - | **0.70 (0.54-0.92)** | **.010** |
| Relative family affluence (ref. low) |  |  |  |  |
| Medium | **1.79 (1.29-2.48)** | **<.001** | 1.19 (0.95-1.50) | .131 |
| High | 1.26 (0.88-1.81) | .203 | 1.17 (0.90-1.52) | .243 |
| Family structure (ref. nuclear family) |  |  |  |  |
| Single-parent family | - | - | - | - |
| Stepfamily | - | - | - | - |
| Urban residence (ref. rural) | - | - | - | - |
| Immigrant background (ref. native) |  |  |  |  |
| First-generation immigrant | **0.57 (0.34-0.96)** | **.034** | **0.49 (0.34-0.72)** | **<.001** |
| Second-generation immigrant | 0.80 (0.50-1.28) | .345 | **0.62 (0.46-0.84)** | **.002** |

*Note.* Adjusted mixed-effect multinomial logistic regression models per sample: odds ratios (OR), 95% confidence intervals (CI), ref. reference category. Dependent variable = respondents excluded or included in cluster analysis (1 = excluded, 2 = included).

- Not tested in the adjusted model due to non-significant (*p* > .05) univariate association.

Additional file 1: **Table S4.** School-level variance in mixed effect multinomial logistic regression analysis models.

| Variable |  | Sample 1 (2018) Profile (reference: **Profile 1 – ‘Good mental health’**) | | | | | | Sample 2 (2022) Profile (reference: **Profile 1 – ‘Good mental health’**) | | | | | |
| --- | --- | --- | --- | --- | --- | --- | --- | --- | --- | --- | --- | --- | --- |
|  |  | **Profile 2 – ‘Poor mental health’** | | **Profile 3 – ‘Moderate mental health’** | | **Profile 4 – 'Somatically troubled'** | | **Profile 2 – ‘Poor mental health’** | | **Profile 3 – ‘Moderate mental health’** | | **Profile 4 – 'Somatically troubled'** | |
|  |  | School-level variance (*CI* 95%) | *p*-value | School-level variance (*CI* 95%) | *p*-value | School-level variance (*CI* 95%) | *p*-value | School-level variance (*CI* 95%) | *p*-value | School-level variance (*CI* 95%) | *p*-value | School-level variance (*C*  95%) | *p*-value |
| Crude models |  |  |  |  |  |  |  |  |  |  |  |  |  |
| Gender, female (ref. male) | Crude | 0.05 (0.01-0.19) | .147 | 0.07 (0.02-0.20) | .060 | 0.01 (0.00-1.81) | .693 | **0.13 (0.06-0.27)** | **.007** | 0.08 (0.02-0.30) | .139 | **0.25 (0.12-0.53)** | **.008** |
| Grade (ref. 5) | Crude | 0.04 (0.01-0.18) | .224 | 0.07 (0.02-0.19) | .070 | 0.00 | - | **0.09 (0.03-0.22)** | **.039** | 0.07 (0.02-0.31) | .167 | **0.14 (0.06-0.36)** | **.035** |
| Language of instruction, Swedish (ref. Finnish) | Crude | 0.05 (0.01-0.18) | .124 | 0.06 (0.02-0.19) | .092 | 0.00 | - | **0.09 (0.04-0.23)** | **.029** | 0.06 (0.01-0.34) | .275 | **0.28 (0.14-0.54)** | **.003** |
| Relative family affluence (ref. low) | Crude | 0.05 (0.01-0.18) | .140 | 0.07 (0.02-0.19) | .070 | 0.01 (0.00-744.98) | .869 | **0.11 (0.05-0.24)** | **.009** | 0.08 (0.02-0.31) | .160 | **0.28 (0.15-0.54)** | **.003** |
| Family structure (ref. nuclear family) | Crude | 0.05 (0.01-0.18) | .139 | 0.06 (0.02-0.20) | .085 | 0.02 (0.00-0.52) | .540 | **0.14 (0.07-0.27)** | **.005** | 0.06 (0.01-0-33) | .234 | **0.25 (0.12-0.52)** | **.007** |
| Urban residence (ref. rural) | Crude | 0.04 (0.01-0.18) | .171 | 0.06 (0.02-0.19) | .102 |  |  | **0.12 (0.06-0.24)** | **.008** | 0.07 (0.02-0.29) | .166 | **0.28 (0.15-0.55)** | **.003** |
| Immigrant background (ref. native) | Crude | 0.04 (0.01-0.18) | .172 | 0.07 (0.03-0.20) | .060 | 0.02 (0.00-0.85) | .618 | **0.13 (0.06-0.26)** | **.006** | 0.08 (0.02-0.30) | .131 | **0.26 (0.13-0.53)** | **.005** |
| Perceived home atmosphere | Crude | 0.02 (0.00-0.27) | .415 | 0.06 (0.02-0.19) | .096 | 0.01 (0.00-4.39) | .746 | **0.09 (0.04-0.21)** | **.028** | 0.07 (0.01-0.31) | .208 | **0.22 (0.10-0.47)** | **.011** |
|  | Adjusted **a** | 0.03 (0.00-0.27) | .380 | 0.06 (0.02-0.21) | .103 | 0.00 | - | 0.05 (0.01-0.32) | .263 | 0.05 (0.01-0.34) | .281 | 0.09 (0.02-0.44) | .231 |
| Maternal monitoring **^1^** | Crude | 0.02 (0.00-2.80) | .706 | 0.08 (0.02-0.26) | .117 | 0.00 | - | 0.10 (0.04-0.31) | .073 | 0.08 (0.02-0.42) | .229 | 0.18 (0.06-0.50) | .060 |
|  | Adjusted **a** | 0.05 (0.01-0.38) | .353 | 0.09 (0.03-0.29) | .102 | 0.00 | - | 0.06 (0.01-0.39) | .294 | 0.09 (0.02-0.47) | .232 | 0.08 (0.01-0.58) | .309 |
| Paternal monitoring **^1^** | Crude | 0.00 | - | 0.06 (0.02-0.26) | .171 | 0.00 | - | 0.11 (0.03-0.32) | .078 | 0.09 (0.02-0.44) | .216 | 0.13 (0.04-0.44) | .111 |
|  | Adjusted **a** | 0.04 (0.00-0.45) | .413 | 0.07 (0.02-0.30) | .192 | 0.00 | - | 0.02 (0.00-7.84) | .750 | 0.09 (0.02-0.50) | .238 | 0.10 (0.02-0.51) | .216 |
| Family support | Crude | 0.04 (0.01-0.19) | .205 | 0.06 (0.02-0.19) | .095 | 0.02 (0.00-1.00) | .638 | **0.11 (0.05-0.23)** | **.010** | 0.08 (0.02-0.30) | .135 | **0.20 (0.09-0.45)** | **.015** |
|  | Adjusted **a** | 0.04 (0.01-0.22) | .236 | 0.07 (0.02-0.21) | .095 | 0.00 | - | 0.06 (0.01-0.29) | .192 | 0.06 (0.01-0.32) | .219 | 0.07 (0.01-0.45) | .290 |
| Peer support | Crude | 0.04 (0.01-0.18) | .180 | 0.07 (0.03-0.20) | .053 | 0.02 (0.00-0.95) | .632 | **0.12 (0.06-0.25)** | **.006** | 0.08 (0.02-0.31) | .131 | **0.28 (0.14-0.55)** | **.004** |
|  | Adjusted **a** | 0.04 (0.01-0.23) | .293 | 0.08 (0.03-0.22) | .057 | 0.00 | - | 0.07 (0.02-0.29) | .145 | 0.07 (0.02-0.32) | .195 | 0.08 (0.01-0.44) | .269 |
| Teacher support | Crude | 0.06 (0.02-0.19) | .100 | 0.06 (0.02-0.19) | .099 | 0.01 (0.00-26.04) | .808 | **0.10 (0.04-0.22)** | **.014** | 0.09 (0.03-0.32) | .112 | **0.17 (0.07-0.41)** | **.028** |
|  | Adjusted **a** | 0.06 (0.02-0.21) | .108 | 0.06 (0.02-0.21) | .100 | 0.00 | - | 0.05 (0.01-0.35) | .330 | 0.07 (0.02-0.33) | .193 | 0.08 (0.01-0.46) | .277 |
| Classmate support | Crude | 0.04 (0.01-0.19) | .210 | 0.06 (0.02-0.20) | .078 | 0.01 (0.00-34.14) | .817 | **0.13 (0.06-0.26)** | **.005** | 0.09 (0.02-0.32) | .129 | **0.22 (0.10-0.50)** | **.016** |
|  | Adjusted **a** | 0.04 (0.01-0.23) | .294 | 0.07 (0.02-0.21) | .083 | 0.00 | - | 0.08 (0.02-0.29) | .145 | 0.07 (0.01-0.35) | .221 | 0.08 (0.01-0.48) | .279 |
| Perceived school climate | Crude | 0.01 (0.00-1.33) | .675 | 0.06 (0.02-0.19) | .092 | 0.01 (0.00-20.79) | .804 | **0.10 (0.05-0.22)** | **.013** | 0.08 (0.02-0.31) | .165 | **0.22 (0.10-0.52)** | **.020** |
|  | Adjusted **a** | 0.02 (0.00-0.56) | .561 | 0.06 (0.02-0.21) | .094 | 0.00 | - | 0.05 (0.01-0.30) | .261 | 0.06 (0.01-0.33) | .245 | 0.09 (0.02-0.50) | .251 |
| Academic educational expectations **^2^** (ref. vocational) | Crude | 0.05 (0.00-0.98) | .522 | 0.03 (0.00-3.91) | .688 | 0.00 | - | 0.02 (0.00-11.23) | .761 | 0.00 | - | 0.27 (0.09-0.83) | .076 |
|  | Adjusted **a** | 0.06 (0.00-0.99) | .484 | 0.05 (0.00-1.42) | .567 | 0.00 | - | 0.00 | - | 0.00 | - | 0.13 (0.02-1.03) | .339 |
| Intensity of online communication | Crude | 0.05 (0.02-0.20) | .133 | **0.08 (0.03-0.21)** | **.046** | 0.00 | - | **0.11 (0.05-0.24)** | **.016** | 0.04 (0.01-0.40) | .369 | **0.22 (0.10-0.50)** | **.016** |
|  | Adjusted **a** | 0.05 (0.01-0.23) | .209 | 0.08 (0.03-0.22) | .060 | 0.00 | - | 0.07 (0.02-0.31) | .175 | 0.03 (0.00-0.45) | .444 | 0.12 (0.03-0.49) | .158 |
| Health literacy **1** (ref. low) | Crude | 0.02 (0.00-2.36) | .699 | 0.07 (0.02-0.27) | .154 | 0.00 | - | 0.08 (0.02-0.28) | .124 | 0.07 (0.01-0.41) | .251 | **0.19 (0.08-0.48)** | **.034** |
|  | Adjusted **a** | 0.03 (0.00-0.51) | .482 | 0.08 (0.02-0.28) | .111 | 0.00 | - | 0.04 (0.00-0.50) | .444 | 0.09 (0.02-0.47) | .249 | 0.10 (0.02-0.52) | .230 |
| Self-rated health (ref. poor) | Crude | 0.03 (0.01-0.21) | .282 | 0.05 (0.01-0.19) | .179 | 0.01 (0.00-5.93) | .759 | **0.10 (0.04-0.24)** | **.020** | 0.08 (0.02-0.31) | .165 | **0.23 (0.11-0.48)** | **.008** |
|  | Adjusted **a** | 0.04 (0.01-0.22) | .232 | 0.05 (0.01-0.21) | .183 | 0.01 (0.00-46.74) | .822 | 0.7 (0.02-0.31) | .180 | 0.06 (0.01-0.34) | .268 | 0.09 (0.02-0.41) | .205 |
|  |  |  |  |  |  |  |  |  |  |  |  |  |  |
| Adjusted models |  |  |  |  |  |  |  |  |  |  |  |  |  |
| Adjusted model **a**, all socio-demographic and social characteristics included |  | 0.04 (0.01-0.20) | .223 | 0.07 (0.02-0.21) | .094 | 0.00 | - | 0.07 (0.02-0.29) | .153 | 0.06 (0.01-0.33) | .250 | 0.08 (0.02-0.40) | .226 |
| Adjusted model **b** |  | 0.05 (0.01-0.33) | .314 | 0.08 (0.03-0.24) | .067 | 0.01 (0.00-3.39) | .722 | 0.03 (0.00-0.81) | .533 | 0.04 (0.00-0.47) | .424 | 0.18 (0.04-0.73) | .162 |
| Adjusted model **c^1^** |  | 0.09 (0.02-0.44) | .226 | 0.08 (0.02-0.34) | .174 | 0.01 (0.00-305.57) | .849 | 0.03 (0.00-3.20) | .683 | 0.08 (0.01-0.63) | .330 | 0.25 (0.07-0.95) | .142 |
| Adjusted model **d^2^** |  | 0.07 (0.00-2.55) | .586 | 0.13 (0.02-0.80) | .291 | 0.00 | - | 0.00 | - | 0.02 (0.00-23.49) | .784 | 0.00 | - |

*Note.* Mixed-effect multinomial logistic regression models per profile: odds ratios (OR), 95% confidence intervals (CI), ref. reference category.

**^1^** Only 7^th^ and 9^th^ grade adolescents included.

**^2^** Only 9^th^ grade adolescents included.

**a** adjusted for socio-demographic characteristics (gender, grade level, language of instruction, relative family affluence, family structure, urban/rural residence, immigrant background).

**b** adjusted for socio-demographic characteristics, psychosocial factors (perceived social support, perceived home atmosphere, frequency of online communication, perceptions of school cohesion), and self-rated health.

**c** adjusted for socio-demographic characteristics, psychosocial factors (perceived social support, perceived home atmosphere, frequency of online communication, perceptions of school cohesion, parental monitoring), and other health-related factors (self-rated health and health literacy).

**d** adjusted for socio-demographic characteristics, psychosocial factors (perceived social support, perceived home atmosphere, frequency of online communication, perceptions of school cohesion, parental monitoring), and other health-related factors (self-rated health, health literacy, and educational expectations).
